# B cell deficiency induces cytotoxic memory CD8^+^ T cells during influenza-associated bacterial pneumonia

**DOI:** 10.1172/JCI188342

**Published:** 2025-06-10

**Authors:** Leigh M. Miller, Alexis M. Duray, Ellyse M. Cipolla, Flavia Rago, Brooke P. Dresden, Kristen L. Parenteau, Abhigya Gupta, John F. Alcorn

**Affiliations:** 1 Department of Pediatrics, UPMC Children’s Hospital of Pittsburgh, Pittsburgh, Pennsylvania, USA.; 2 Dietrich School of Arts and Sciences and; 3 Department of Immunology, University of Pittsburgh, Pittsburgh, Pennsylvania, USA.

**Keywords:** Immunology, Infectious disease, Adaptive immunity, Influenza, T cells

## Abstract

Influenza-associated bacterial superinfections in the lung lead to increased morbidity and mortality. Nearly all people have preexisting memory to influenza virus, which can protect against subsequent infection in the lung. This study explored the role B cells play in protection against bacterial (*Staphylococcus aureus* or *Klebsiella pneumoniae*) superinfection with previous heterotypic influenza memory. B cell deficiency resulted in an increased inflammatory lung environment and lung tissue injury during superinfection. Loss of B cells increased populations of memory CD8^+^ T cells in the lung, and these CD8^+^ T cells were transcriptionally and functionally distinct from those of WT mice. Use of antibody-deficient mouse models showed that this phenotype was specifically due to loss of antibody production from B cells. Passive immunization with influenza antibody serum in B cell–deficient mice rescued the CD8^+^ T cell phenotype. CD8^+^ T cell depletion and lethal superinfection challenge experiments showed that the cytotoxic memory CD8^+^ T cells from B cell–deficient mice protect against superinfection bacterial burden and mortality. These findings provide insight into the importance of B cells for regulating immune responses against infection.

## Abstract 2

B cell antibody production impacts memory CD8+ T cell immunity during influenza, Staphylococcus aureus super-infection.

## Graphical abstract

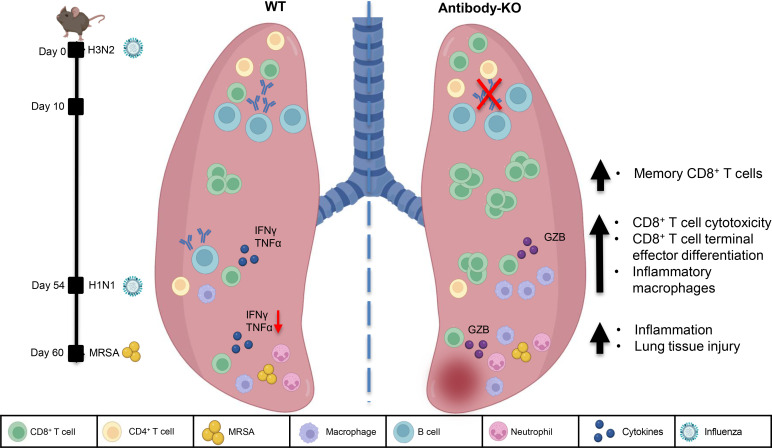

## Introduction

Seasonal influenza annually contributes to significant morbidity and mortality among elderly, young, and immunocompromised persons. Influenza virus infection results in an inflammatory environment in which the lung epithelium becomes damaged (1, 2). This process causes dysregulation of innate and adaptive immunity, leading to increased susceptibility to secondary bacterial pneumonias (3–6). Secondary bacterial pneumonias, particularly those caused by methicillin-resistant *Staphylococcus aureus* (MRSA), result in increased morbidity and mortality both during seasonal influenza seasons and during pandemics, including those of 1918 and 2009 (7). Lung tissue impairment and long-term complications can occur (8). Mouse models have shown that immunity to bacterial infection in the lung is impaired by preceding antiviral responses (9–16). Immune dysregulation by viral infection causes increased bacterial burden in the lung during superinfection, leading to increased lung inflammation and tissue injury in comparison with single infection (17, 18). It is critical to develop an understanding of how immune mechanisms become dysregulated during influenza-associated bacterial pneumonias.

Following clearance of influenza virus, innate and adaptive immune systems form memory. Influenza virus is coated with surface antigens hemagglutinin (HA) and neuraminidase (NA). Memory B cells primarily secrete neutralizing antibodies targeted against HA and NA (19–21). However, antigenic drift of HA and NA decreases the efficiency of the preexisting antibody repertoire over time, rendering pre-formed antibodies ineffective against heterotypic influenza virus infection (19–21). Lack of cross-protective antibodies against influenza virus is a primary reason why seasonal influenza vaccines need to be reformulated annually (19–21). Influenza viruses also contain conserved internal antigens, such as nucleoprotein (NP), that are less susceptible to mutating (21, 22). Memory CD4^+^ and CD8^+^ T cells recognize these internal viral epitopes, making them highly effective at providing cross-protective responses against heterotypic influenza viruses (21–25). Following clearance of influenza virus, T cells form memory subsets, such as central memory, effector memory, and resident memory. Lung tissue-resident memory T cells (TRMs) have been shown to provide optimal protection against influenza virus and are critical for providing heterosubtypic immunity. CD8^+^ TRMs primarily act to detect reinfection at sites of pathogen entry and secrete cytokines, such as IFN-γ, TNF-α, and granzymes, to mediate antiviral immunity (23–27). However, cytotoxic memory CD8^+^ T cells may act as a double-edged sword; CD8^+^ T cell activation is essential for viral and bacterial clearance but may also contribute to increased tissue damage (28–30).

A majority of studies relating to influenza-associated bacterial infections focus on acute disease in previously naive mice; however, nearly all humans have preexisting immunity to influenza virus, and repeat infection with the same influenza virus strain is uncommon (31). Therefore, an influenza-associated bacterial pneumonia model that takes preexisting heterotypic influenza memory into account may be more clinically relevant. Our group previously showed that prior challenge with heterosubtypic influenza virus in mice improves control of subsequent superinfection and reduces lung tissue injury (32). Because lung memory B and T cells are key arms of preexisting immunity, we explored the functional role of B cells for heterotypic protection. Using spectral flow cytometry and transcriptomics, we studied the importance of B cells for clearance of pathogen and prevention of tissue injury in the lung. B cells were manipulated using genetic and temporal antibody depletion and passive transfer of influenza antibody serum. These data demonstrate the functional role of B cells in heterotypic influenza virus strain immunity and how loss of B cell function impacts formation of lung memory CD8^+^ T cells and exacerbates lung inflammation following heterotypic influenza virus strain superinfection.

## Results

### MRSA challenge following heterotypic influenza memory increases tissue injury in the lung and alters the innate cell compartment.

**Figure 1:**
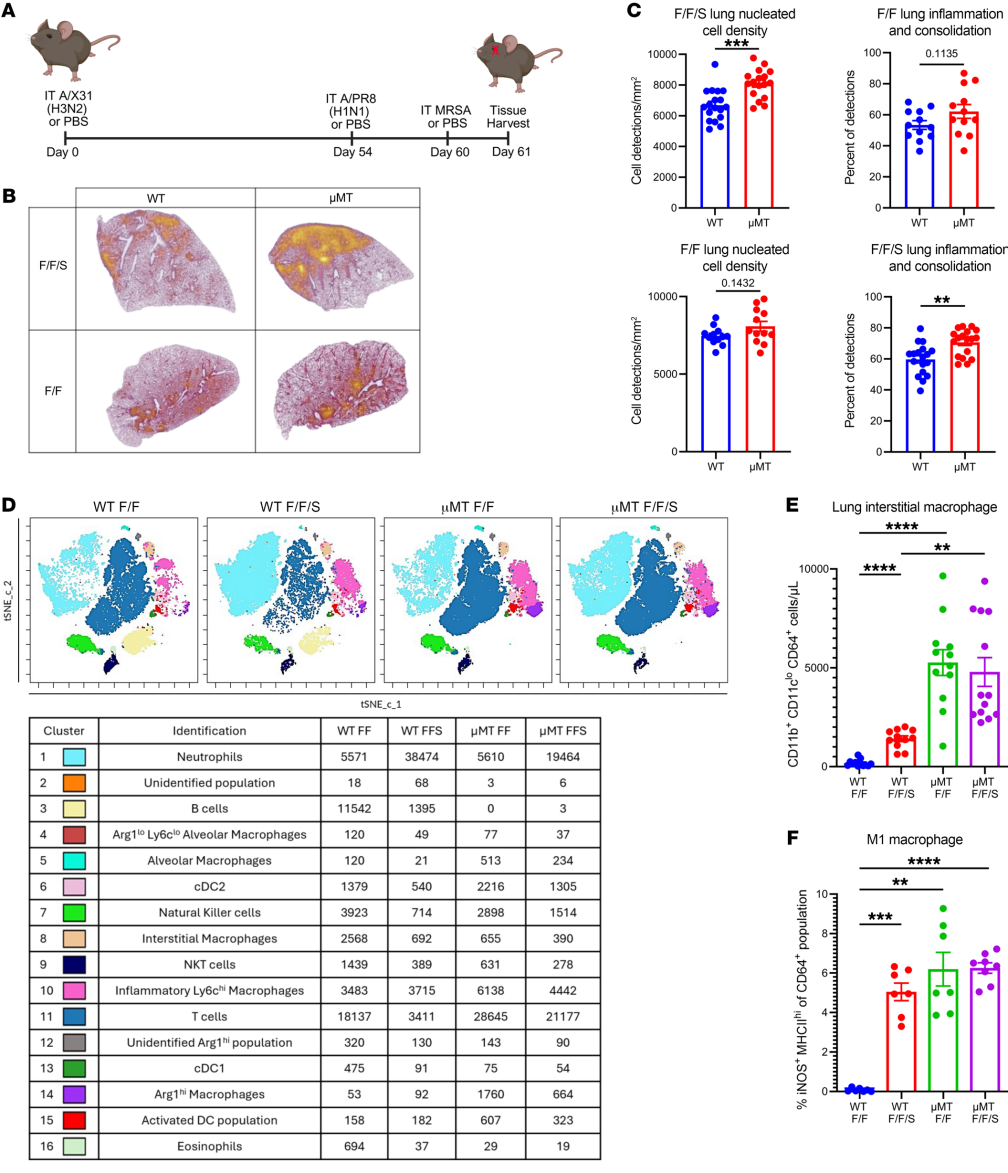
Loss of B cells increases lung populations of T cells and inflammatory macrophages during memory superinfection. (**A**) Infection scheme for memory-superinfected mice. IT, intratracheal. (**B**) Representative H&E lung images (original magnification, ×40) with a heatmap overlay to signify lung areas with inflammation/consolidation. (**C**) Lung nucleated cell density (cells/mm^2^) and frequency of lung inflammation and consolidation detections between WT and μMT lungs and between F/F and F/F/S treatment groups (WT-F/F: *n* = 12; WT-F/F/S: *n* = 18; μMT-F/F: *n* = 12; μMT-F/F/S: *n* = 18). (**D**) Flow cytometry analysis was conducted on WT and μMT mice with F/F and F/F/S infections. Samples were concatenated (*n* = 4), and populations were visualized by FlowSOM and t-SNE–CUDA using Cytobank software. (**E** and **F**) Samples were analyzed by flow cytometry. Absolute number of CD64^+^CD11b^+^CD11c^lo^ cells (WT-F/F: *n* = 12; WT-F/F/S: *n* = 11; μMT-F/F: *n* = 12; μMT-F/F/S: *n* = 13) and percentage iNOS^+^MHCII^hi^ cells of CD64^+^ cells (WT-F/F: *n* = 7; WT-F/F/S: *n* = 8; μMT-F/F: *n* = 7; μMT-F/F/S: *n* = 8). Data are represented as mean ± SEM, and *P* values were determined by repeated 1-way ANOVA measures (**P < 0.01, ***P <0.001, ****P < 0.0001).

To investigate how B cells impact immune memory during superinfection, we primed WT or B cell–deficient (μMT) mice with influenza A/HKx31 (H3N2, X-31) virus on day 0, followed by rechallenge with a heterotypic strain of influenza A/PR/8/34 (H1N1, PR8) virus at day 54. Six days after PR8 challenge, mice were inoculated with MRSA USA300 or PBS, and tissues were harvested 24 hours later (Figure 1A). We used mouse-adapted strains X-31 and PR8 as they have similar internal proteins while expressing dissimilar proteins on their surface. These influenza viruses have been used previously by others, and this preexisting influenza memory superinfection model (Figure 1A) has been characterized by our group (32–34). Prior experiments examined how the addition of heterotypic influenza memory impacted control of superinfection (F/F/S) compared with acute infection (F/S). WT mice with F/F/S infection lost significantly less weight compared with WT mice with F/S infection, but heterotypic memory did not impact weight loss in μMT mice (Supplemental Figure 1A; supplemental material available online with this article; https://doi.org/10.1172/JCI188342DS1) (32). Interestingly, both WT and μMT mice with F/F/S infection had lower lung MRSA burden and viral burden compared with their F/S controls (Supplemental Figure 1, B and C). Because bacterial and viral burden was not significantly different between WT and μMT mice during F/S or F/F/S infection, we investigated whether lung tissue injury was different. We measured lung tissue injury using 2 methods: sample-blinded scoring by investigators; and QuPath, which allowed us to measure immune cell infiltration and lung tissue inflammation using machine learning–based algorithms. Comparing lung tissue injury in F/S mice versus F/F/S mice showed that there was no significant change in lung tissue injury between WT and μMT mice during F/S infection (Supplemental Figure 1D). However, WT mice had improved control of parenchymal tissue injury during F/F/S infection, but this was not the case with μMT mice, which had significantly more parenchymal tissue injury compared with WT mice (Supplemental Figure 1D).

To further assess the impact of MRSA challenge on the lung inflammatory environment during B cell deficiency, we compared lung tissue injury in WT and μMT mice during memory heterotypic influenza virus (F/F) or memory superinfection (F/F/S). Using QuPath, mice with F/F infection trended toward decreased lung inflammation and consolidation (dense areas with no space for air exchange) compared with F/F/S regardless of genotype, but this finding was not statistically significant (Supplemental Figure 2A). We then assessed tissue integrity by measuring protein leak into airspaces and found significantly higher levels of protein in the bronchoalveolar lavage fluid (BALF) of WT and μMT mice that received F/F/S infection compared with mice with F/F infection (Supplemental Figure 2B). Using QuPath, we overlaid a heatmap highlighting areas of inflammation and consolidation; this showed that WT and μMT mice had more lung inflammation during F/F/S infection compared with F/F infection (Figure 1B). B cell deficiency trended toward a moderate increase in cell density and inflammation in the lung during F/F infection compared with WT, but these data were not statistically significant. However, we found that during F/F/S infection, B cell deficiency significantly increased lung tissue injury and cell density in the lung in comparison with WT mice (Figure 1C).

Next, we explored how MRSA challenge altered the lung immune cell compartment with heterotypic memory influenza virus infection in WT and μMT mice. Using conventional flow cytometry gating (Supplemental Figure 3) and dimensionality reduction (t-distributed stochastic neighbor embedding [t-SNE]–CUDA) and clustering (FlowSOM) (both from Cytobank [Beckman Coulter]), we tracked changes of the innate and adaptive immune cell compartments in lungs (Figure 1D). We observed larger populations of T cells and macrophages (interstitial and exudate) in the lungs of μMT compared with WT mice (Figure 1, D and E, and Supplemental Figure 2C). Additionally, we found greater numbers of both M1-like (iNOS^+^MHCII^hi^) and M2-like (CD206^+^) macrophages in the lungs of μMT compared with WT mice (Figure 1F and Supplemental Figure 2D). We also found more NK cells in the lungs of μMT mice compared with WT (Supplemental Figure 2E). We found that the largest change in the immune cell compartment of F/F versus F/F/S mice following MRSA challenge was an increase in the number of neutrophils (Figure 1D and Supplemental Figure 2F). While the number of neutrophils did not change between WT and μMT lungs, the neutrophils of μMT lungs were proportionally more mature and expressed higher levels of inducible nitric oxide synthase (iNOS), a key regulator of neutrophil function (Supplemental Figure 2, G and H). Additionally, we observed an increase in genes related to neutrophil function in WT mice during F/F/S challenge compared with F/F challenge with gene expression unchanged between μMT F/F and F/F/S infection (Supplemental Figure 2I). We saw that the number of B cells in the lung tissue of WT mice significantly decreased following MRSA challenge (Figure 1D and Supplemental Figure 2J). We additionally compared viral burden between WT and μMT F/F and F/F/S groups and observed no significant differences between infection groups (Supplemental Figure 2K). Next, we compared changes in inflammatory cytokines/mediators in the lungs of both WT and μMT mice. For both WT and μMT mice, we observed increases in some proinflammatory cytokines (IL-1α, IL-1β, IL-6, IL-12p40) and other mediators that promote neutrophil chemotaxis to the lung (G-CSF, KC/CXCL1) following MRSA challenge (Supplemental Figure 2L). Unlike the lungs of μMT mice, the lungs of WT mice had significantly higher expression of mediators that promote macrophage migration (MCP-1, MIP-1α, MIP-1β), which may reflect the higher number of macrophages already present in the lungs of μMT mice prior to MRSA challenge (Figure 1E and Supplemental Figure 2C). Another interesting finding was that IL-10 was decreased in the lungs of both WT and μMT mice following MRSA challenge (Supplemental Figure 2L). Together these data show that MRSA challenge altered the lung environment of both WT and μMT mice toward a state of higher inflammation, but μMT mice had larger populations of T cells and macrophages in the lung compared with WT during F/F and F/F/S infection.

### Absence of B cells increases formation of cytotoxic effector CD8^+^ T cells in the lung.

**Figure 2:**
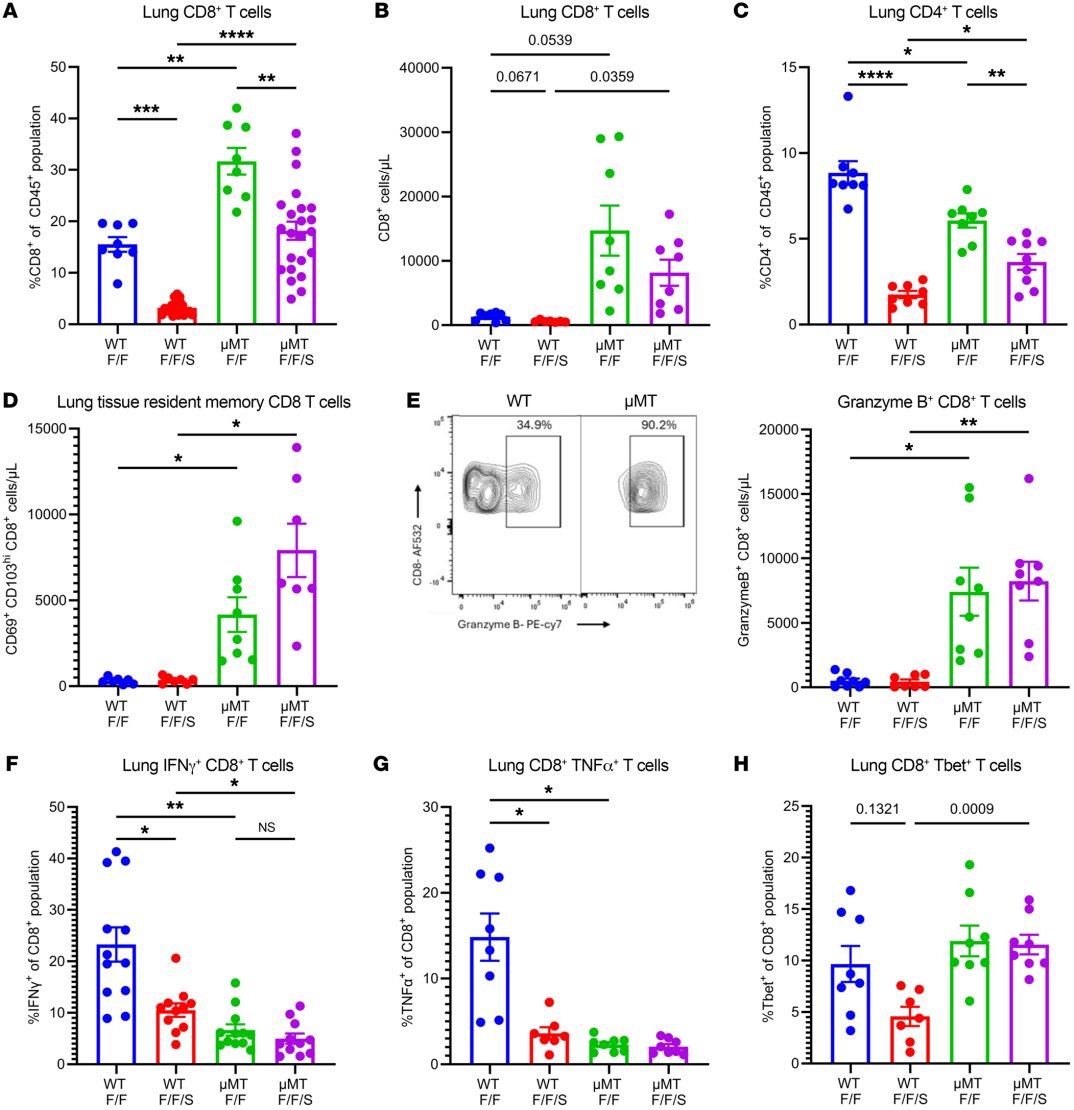
Loss of B cells alters CD8^+^ T cell number and phenotype during memory superinfection. Flow cytometry analysis was conducted on WT and μMT mice with F/F and F/F/S infections. (**A** and **B**) Percentage (WT-F/F: *n* = 8; WT-F/F/S: *n* = 20; μMT-F/F: *n* = 8; μMT-F/F/S: *n* = 24) and absolute number (WT-F/F: *n* = 8; WT-F/F/S: *n* = 7; μMT-F/F: *n* = 8; μMT-F/F/S: *n* = 8) of CD90.2^+^CD8^+^ cells. (**C**) Percentage of CD4^+^CD90.2^+^ cells (WT-F/F: *n* = 8; WT-F/F/S: *n* = 7; μMT-F/F: *n* = 8; μMT-F/F/S: *n* = 8). (**D**) Absolute number of CD8^+^CD69^+^CD103^hi^ cells (WT-F/F: *n* = 8; WT-F/F/S: *n* = 7; μMT-F/F: *n* = 8; μMT-F/F/S: *n* = 8). (**E**) Intracellular flow cytometry plot (left) showing the percentage granzyme B^+^CD8^+^ T cells using concatenated F/F/S infected WT and μMT samples (WT-F/F/S: *n* = 4; μMT-F/F/S: *n* = 4) and absolute number of granzyme B^+^CD8^+^ T cells (right) (WT-F/F: *n* = 8; WT-F/F/S: *n* = 7; μMT-F/F: *n* = 8; μMT-F/F/S: *n* = 8). (**F**–**H**) Percentage of IFN-γ^+^CD8^+^ cells (WT-F/F: *n* = 12; WT-F/F/S: *n* = 11; μMT-F/F: *n* = 12; μMT-F/F/S: *n* = 11), TNF-α^+^CD8^+^ cells, and Tbet^+^CD8^+^ cells (WT-F/F: *n* = 8; WT-F/F/S: *n* = 7; μMT-F/F: *n* = 8; μMT-F/F/S: *n* = 8). Data are represented as mean ± SEM, and *P* values were determined by repeated 1-way ANOVA measures (*P < 0.05, **P < 0.01, ***P <0.001, ****P < 0.0001).

It has been shown by others that heterotypic strain control of influenza virus is largely T cell dependent (22, 23). To further characterize the changes in the T cell compartment of WT and μMT mice during F/F and F/F/S infection, we examined marker expression changes of T cells in the lung. Using our T cell gating strategy (Supplemental Figure 4), we found that CD4^+^ and CD8^+^ T cells were significantly increased in both number and proportion in the lungs of μMT compared with WT mice, during both F/F infection and F/F/S infection, with CD4^+^ and CD8^+^ T cells decreased in the lung as a result of MRSA challenge (Figure 2, A–C, and Supplemental Figure 5, A–C). Additionally, μMT lungs had significantly higher numbers of effector memory and TRM CD8^+^ T cells compared with WT lungs during both F/F infection and F/F/S infection (Figure 2D and Supplemental Figure 5D). Next, we examined the functional phenotype of these effector CD8^+^ T cells by examining expression of transcription factors and proinflammatory cytokines. We observed that the majority of lung CD8^+^ T cells of μMT mice expressed granzyme B, a marker of cytotoxicity, whereas WT lungs had a lower proportion and absolute number of granzyme B^+^ CD8^+^ T cells (Figure 2E). Next, we looked at expression of key antiviral cytokines, IFN-γ and TNF-α, and found that the lung CD8^+^ T cells of μMT mice had significantly lower expression of these cytokines compared with WT (Figure 2, F and G). However, we did not find lower expression of Tbet in the lung CD8^+^ T cells of μMT, indicating that these CD8^+^ T cells have impaired cytokine production (IFN-γ, TNF-α), but not impaired differentiation into an antiviral effector phenotype (Figure 2, F–H). Interestingly, MRSA challenge in WT mice (F/F/S) significantly decreased the proportion of lung CD8^+^ T cells expressing IFN-γ and TNF-α compared with F/F infection, which further highlights the immune dysfunction caused by secondary MRSA infection. These data show that B cell deficiency increases the population of effector and resident memory CD8^+^ T cells in the lung and alters their functional phenotype.

We next explored whether this phenotype was unique to heterotypic infection by comparing heterotypic influenza superinfection with homotypic influenza (PR8-PR8) superinfection. WT and μMT mice lost more weight during heterotypic infection compared with homotypic infection (Supplemental Figure 6A). While bacterial burden was unchanged between groups, viral burden was increased in μMT mice with heterotypic infection compared with homotypic infection (Supplemental Figure 6, B and C). For both WT and μMT mice, no significant differences were observed in the innate immune cells (neutrophils, macrophages) (Supplemental Figure 6, D and E). Heterotypic influenza infection resulted in an increase in effector memory and TRM CD8^+^ T cells in the lungs of both WT and μMT mice compared with homotypic infection (Supplemental Figure 6, F and G). Interestingly, there was an increase in the proportion of influenza-specific CD8^+^ T cells in WT mice with heterotypic infection compared with homotypic infection, but not in the lungs of μMT mice (Supplemental Figure 6H). For both heterotypic and homotypic influenza infections, CD8^+^ T cells of μMT mice had higher coexpression of PD-1, LAG-3, and TIM-3 compared with WT; however, this coexpression was greater in heterotypic infection compared with homotypic infection for both WT and μMT mice (Supplemental Figure 6I). Additionally, granzyme B concentration in the BALF was increased with heterotypic infection compared with homotypic infection for WT and μMT mice (Supplemental Figure 6J). Lung tissue injury was increased in heterotypic infection compared with homotypic infection in WT mice, but this finding was not statistically significant for μMT mice (Supplemental Figure 6K). Overall, these data show that the unique CD8^+^ T cell phenotype of B cell–deficient mice is still observed in homotypic influenza infection, but the phenotype is more pronounced in heterotypic influenza infection.

### Lung CD8^+^ T cells from B cell–deficient mice have an altered transcriptional profile.

**Figure 3:**
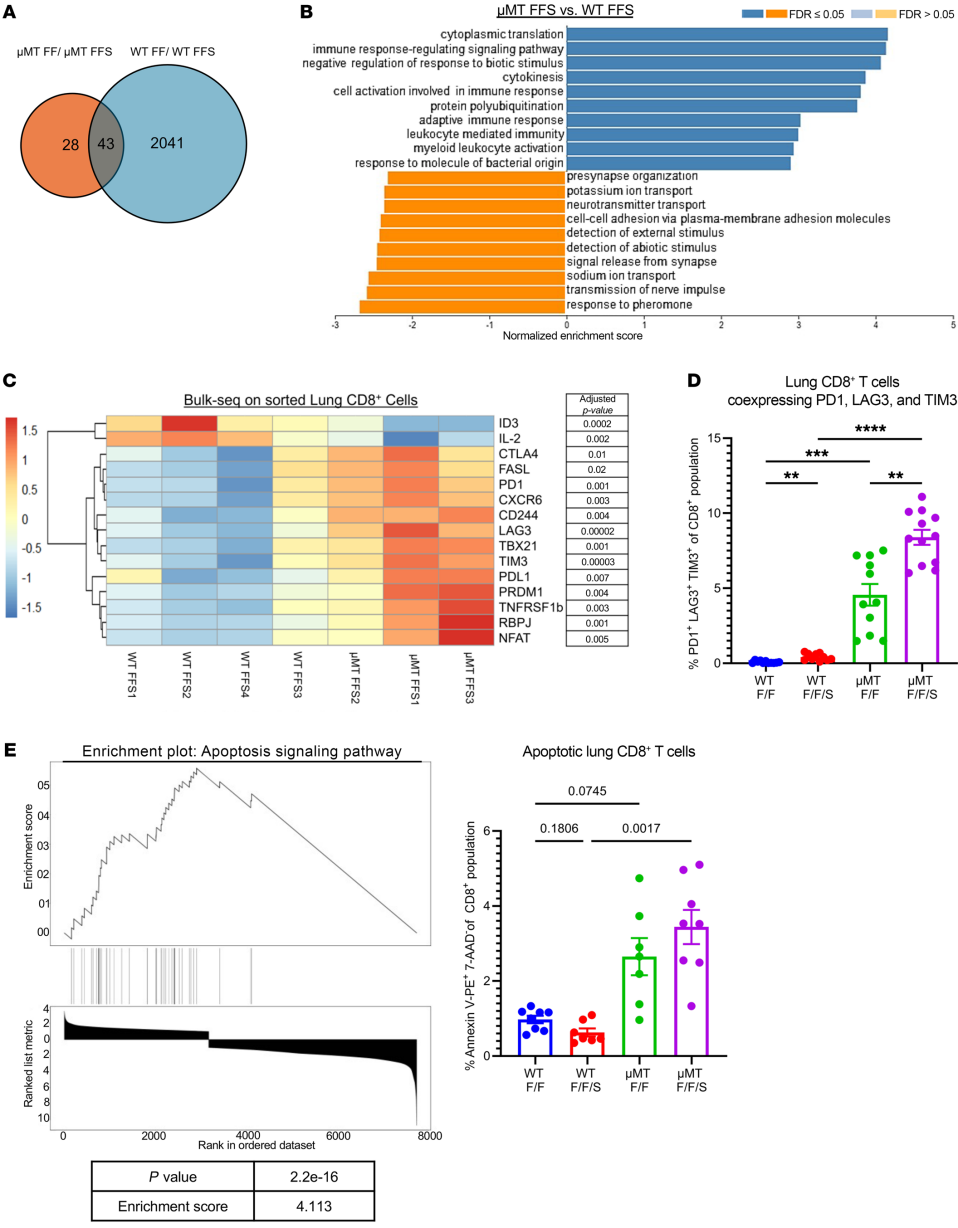
Loss of B cells alters the transcriptional state of lung CD8^+^ T cells during memory superinfection. Bulk RNA sequencing was performed on lung CD8^+^ T cells from WT and μMT mice with either F/F or F/F/S infections (WT-F/F: *n* = 4; WT-F/F/S: *n* = 4; μMT-F/F: *n* = 3; μMT-F/F/S: *n* = 3). (**A**) Venn diagram shows the number of statistically significant differentially expressed genes (DEGs) shared between WT-F/F and WT-F/F/S groups and between μMT-F/F and μMT-F/F/S groups. (**B**) Top and bottom 10 Gene Ontology pathways in μMT-F/F/S versus WT-F/F/S for genes with false discovery rate adjusted *P* values less than 0.05 (8,831 DEGs). (**C**) Clustering heatmap of log_2_-transformed transcripts per million (TPM) values of WT-F/F/S and μMT-F/F/S mice for CD8^+^ T cell genes with adjusted *P* values (right). (**D**) Flow cytometry analysis showing the percentage of PD-1^+^LAG-3^+^TIM-3^+^CD8^+^ T cells (WT-F/F: *n* = 11; WT-F/F/S: *n* = 10; μMT-F/F: *n* = 11; μMT-F/F/S: *n* = 12). (**E**) Gene set enrichment analysis (GSEA) of a pathway in μMT-F/F/S versus WT-F/F/S (top) with *P* value and enrichment score (bottom). Right: Flow cytometry analysis showing the percentage of annexin V^+^7-AAD^–^CD8^+^ T cells (WT-F/F: *n* = 8; WT-F/F/S: *n* = 7; μMT-F/F: *n* = 7; μMT-F/F/S: *n* = 8). Graphed data are represented as mean ± SEM, and *P* values were determined by repeated 1-way ANOVA measures (**P < 0.01, ***P <0.001, ****P < 0.0001).

To further assess the differences in lung CD8^+^ T cells between WT and B cell–deficient (μMT) mice, we sorted the CD8^+^ T cells from the lungs of F/F and F/F/S infected WT and μMT mice and performed bulk RNA sequencing. We examined the sample clustering using principal component analysis and found that the lung CD8^+^ T cells from the WT mice (F/F and F/F/S) had more similarity to each other than to either of the μMT groups (F/F and F/F/S) (Supplemental Figure 7A). We examined how MRSA challenge impacts the transcriptional profile of CD8^+^ T cells in the lung. The CD8^+^ T cells of WT mice had more differentially expressed genes (2,084 genes) between F/F and F/F/S groups compared with μMT lung CD8^+^ T cells, which had fewer differentially expressed genes (71 genes) (Figure 3A). The shared genes (43 genes) between the two were related to cell movement and migration (Supplemental Figure 7B). Interestingly, Gene Ontology pathways and differentially expressed genes associated with immune response signaling, immune cell activation, and control of bacterial infection were downregulated in F/F/S compared with the F/F group in WT mice, suggesting that secondary bacterial challenge in the lung significantly dysregulated immune responses, in particular the type 17 pathway (Supplemental Figure 7, C and D). We looked at the transcriptional differences of lung CD8^+^ T cells between WT and μMT mice during F/F and F/F/S infection (Figure 3B and Supplemental Figure 7, E and F). The pathways enriched in F/F/S μMT mice were associated with adaptive immune response, cell regulation, and response to bacterial stimulus (Figure 3B). We examined transcriptional genes associated with T cell effector function, as well as genes associated with regulation of T cell activation (35–38). We found significant upregulation of several of these markers in μMT mice compared with WT mice during F/F/S infection (Figure 3C). Flow cytometry analysis of lung CD8^+^ T cells coexpressing PD-1, TIM-3, and LAG-3 showed that these markers were significantly upregulated in μMT compared with WT mice and were also increased in proportion upon MRSA challenge (Figure 3D). Intravascular staining further showed that the population of CD8^+^ T cells coexpressing PD-1, TIM-3, and LAG-3 in μMT mice was concentrated in the lung tissue versus the peripheral blood (Supplemental Figure 7G). Gene set enrichment analysis (GSEA) for biological pathways showed that CD8^+^ T cells in μMT lungs were enriched for apoptosis signaling, and flow cytometry analysis confirmed that B cell deficiency during F/F/S infection increased the frequency of apoptotic CD8^+^ T cells in the lung (Figure 3E). Overall, these data show that loss of B cells has a profound impact on the transcriptional profile of lung CD8^+^ T cells, causing upregulation of several genes associated with T cell regulation and effector function.

### Loss of influenza virus–specific antibody increases formation of cytotoxic effector CD8^+^ T cells in the lung during subsequent superinfection.

**Figure 4:**
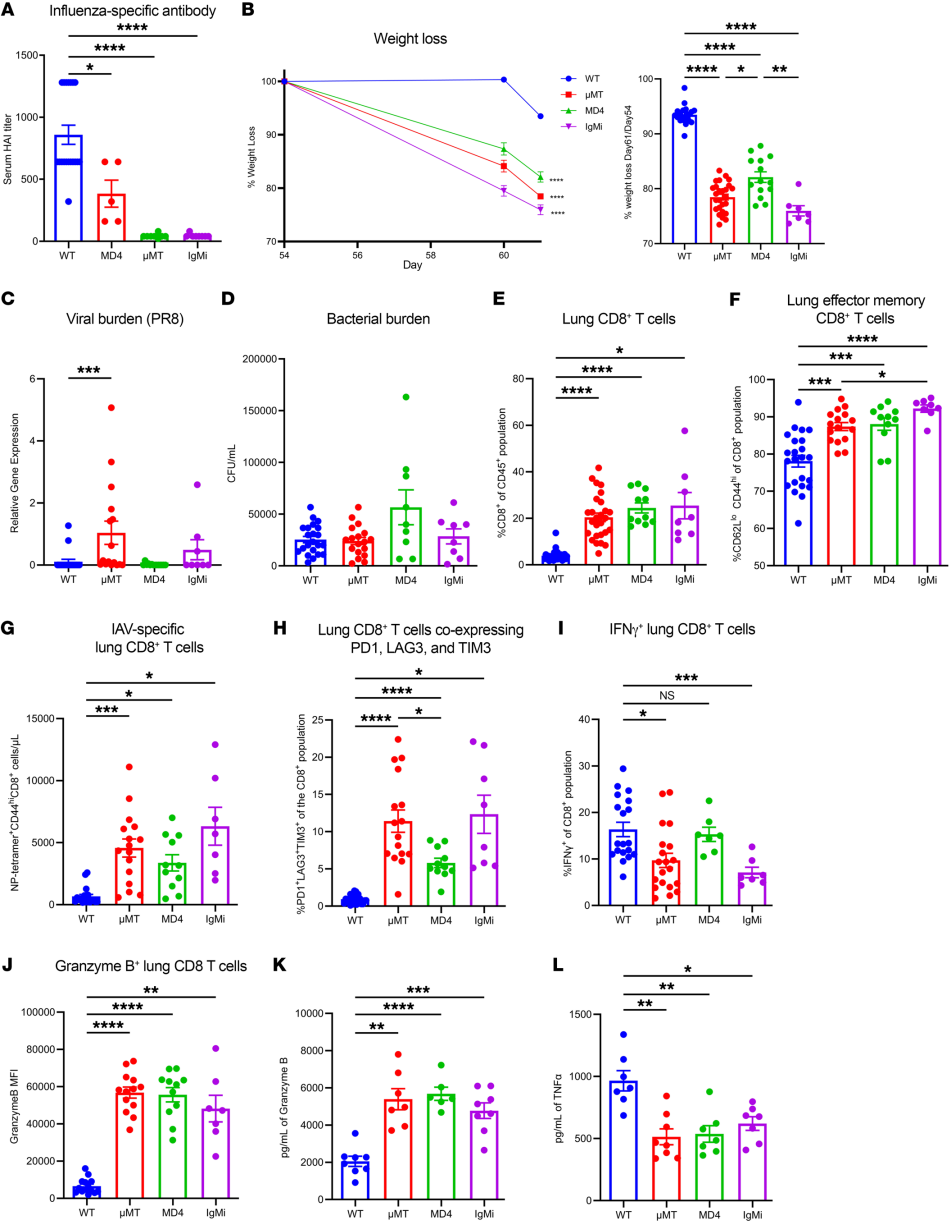
Loss of influenza virus–specific antibody drives cytotoxic memory CD8^+^ T cell responses in superinfection. (**A**) X-31 influenza virus–specific serum titers measured by hemagglutinin inhibition assay (HAI) at day 61 (WT: *n* = 19; MD4: *n* = 5; μMT: *n* = 8; IgMi: *n* = 8). (**B**) Percentage weight loss was calculated starting from PR8 infection (WT: *n* = 18; MD4: *n* = 14; μMT: *n* = 26; IgMi: *n* = 7). (**C**) Viral burden (PR8) assessed via quantitative PCR (WT: *n* = 19; MD4: *n* = 14; μMT: *n* = 16; IgMi: *n* = 8). (**D**) Number of MRSA colonies from lung homogenates (WT: *n* = 22; MD4: *n* = 9; μMT: *n* = 18; IgMi: *n* = 8). (**E**) Percentage of CD8^+^ cells (WT: *n* = 27; MD4: *n* = 11; μMT: *n* = 27; IgMi: *n* = 8). (**F**) Percentage of CD44^hi^CD62^lo^CD8^+^ cells (WT: *n* = 24; MD4: *n* = 11; μMT: *n* = 16; IgMi: *n* = 8). (**G**) Absolute number of NP-tetramer^+^CD44^hi^CD8^+^ cells (WT: *n* = 19; MD4: *n* = 11; μMT: *n* = 16; IgMi: *n* = 8). (**H**) Percentage of PD-1^+^LAG-3^+^TIM-3^+^CD8^+^ cells (WT: *n* = 24; MD4: *n* = 11; μMT: *n* = 16; IgMi: *n* = 8). (**I**) Percentage of IFN-γ^+^CD8^+^ cells (WT: *n* = 19; MD4: *n* = 7; μMT: *n* = 20; IgMi: *n* = 7). (**J**) Median fluorescence intensity (MFI) of granzyme B^+^CD8^+^ T cells (WT: *n* = 14; MD4: *n* = 11; μMT: *n* = 13; IgMi: *n* = 7). (**K** and **L**) Protein expression of granzyme B (WT: *n* = 8; MD4: *n* = 6; μMT: *n* = 7; IgMi: *n* = 8) and TNF-α (WT: *n* = 7; MD4: *n* = 7; μMT: *n* = 8; IgMi: *n* = 7) from BALF. Data are represented as mean ± SEM, and *P* values were determined by repeated 1-way ANOVA measures (*P < 0.05, **P < 0.01, ***P <0.001, ****P < 0.0001).

We sought to determine which B cell function was causing the distinct CD8^+^ T cell phenotype observed in μMT mice. To examine the role of memory B cell antibody secretion during subsequent superinfection, we used 4 different strains of mice: WT C57BL/6 mice, B cell–deficient mice (μMT), MD4 mice, and IgMi mice. MD4 mice, which have a transgenic B cell receptor specific to hen egg lysozyme, produce 10 times fewer virus-specific precursor splenic B cells compared with WT mice (39). During F/F/S infection, MD4 mice produced influenza virus X-31–specific antibody, but at significantly lower levels compared with WT mice (Figure 4A). IgMi mice failed to produce any soluble antibody, similarly to μMT mice, as all constant regions in the IgH chain are deleted, preventing class switching from IgM (40). We showed that MD4 mice represent an intermediate influenza virus–specific antibody–deficient mouse strain in our study, while the IgMi mice are a complete influenza virus–specific antibody knockout (Figure 4A). WT mice lost significantly less weight compared with our B cell–deficient strains (μMT, MD4, and IgMi) with μMT and IgMi mice having lost more weight than the MD4 mice, suggesting that weight loss during F/F/S infection is dependent on the presence of preexisting influenza virus–specific antibody (Figure 4B). While μMT mice had more viral burden in the lung compared with WT mice, a majority of mice in all 4 groups (WT, μMT, MD4, and IgMi) had non-detectable levels of PR8 gene expression (Figure 4C). Interestingly, all 4 groups had similar amounts of bacterial burden, suggesting that bacterial clearance is not antibody dependent (Figure 4D).

We examined T cell populations during F/F/S infection and saw a significant increase of lung CD8^+^ T cells in all 3 B cell–deficient mouse strains (μMT, MD4, and IgMi) (Figure 4E and Supplemental Figure 8A). A majority of these CD8^+^ T cells were effector memory phenotype (Figure 4F). Additionally, we observed a significant increase in lung CD4^+^ T cells (Supplemental Figure 8, B and C). Loss of B cells or influenza virus–specific antibody increased the absolute number of influenza virus–specific and tissue-resident memory (TRM) CD8^+^ T cells in the lung (Figure 4G and Supplemental Figure 8D). Within the CD8^+^ T cell population, we examined surface markers commonly related to T cell regulation (PD-1, LAG-3, TIM-3) and found significantly increased expression in all 3 B cell–deficient mouse strains (μMT, MD4, and IgMi), with lower expression found in MD4 mice (Figure 4H). Next, we analyzed which cytokines the CD8^+^ T cells were producing in response to F/F/S infection and found significantly lower expression of IFN-γ in μMT and IgMi mice (Figure 4I). However, the CD8^+^ T cells of B cell–deficient mice (μMT, MD4, and IgMi) had high expression of the effector molecule granzyme B as well as elevated concentrations of granzyme B in the BALF, suggesting that loss of B cell antibody induces more cytotoxic lung CD8^+^ T cells (Figure 4, J and K). Finally, we examined the repertoire of inflammatory cytokines expressed in WT and B cell–deficient (μMT, MD4, and IgMi) lungs and BALF and found that certain proinflammatory cytokines, such as TNF-α, were decreased in B cell–deficient mice (μMT, MD4, and IgMi) (Figure 4L and Supplemental Figure 8E). However, cytokines that promote activation of the innate immune compartment (IL-6, MCP-1/CCL2, MIP-1β/CCL4, G-CSF) were increased in B cell–deficient mice (μMT, MD4, and IgMi) (Supplemental Figure 8, E and F). These data suggest that while influenza virus–specific antibody is not necessary for control of bacterial superinfection burden, specific loss of influenza virus–specific antibody results in formation of cytotoxic effector memory CD8^+^ T cells in the lung.

### Loss of influenza virus–specific antibody increases lung tissue injury during memory superinfection.

**Figure 5:**
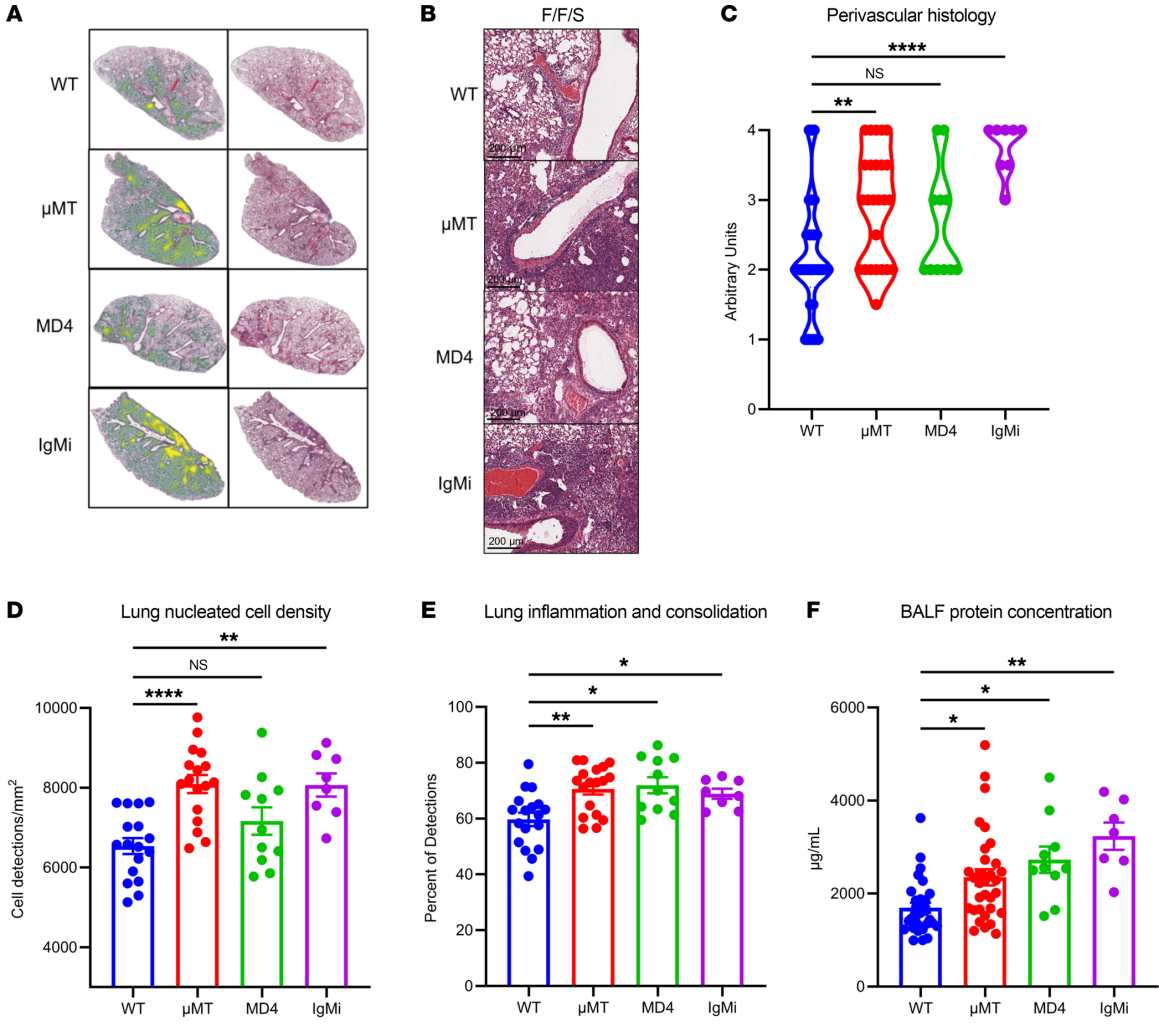
Loss of influenza virus–specific antibody increases lung tissue injury during memory superinfection. Histology scoring and QuPath analysis were performed on lungs from WT, μMT, MD4, and IgMi mice. (**A**) Right: Representative H&E lung images, scanned at ×40. Left: A heatmap overlay was used to signify lung areas with inflammation/consolidation. (**B**) Representative histology sections with H&E of WT, μMT, MD4, and IgMi lungs. Scale bars: 200 μm. (**C**) Histology scores of perivascular lung tissue sections. (**D** and **E**) Quantification of lung nucleated cell density (cells/mm^2^) and frequency of lung inflammation and consolidation detections (WT: *n* = 17; MD4: *n* = 11; μMT: *n* = 17; IgMi: *n* = 8). (**F**) BALF protein at day of harvesting (WT: *n* = 30; MD4: *n* = 10; μMT: *n* = 32; IgMi: *n* = 7). Data are represented as mean ± SEM, and *P* values were determined by repeated 1-way ANOVA measures (*P < 0.05, **P < 0.01, ****P < 0.0001).

A heatmap overlay highlighting areas of inflammation and consolidation showed that lungs of WT mice had inflammation during F/F/S infection, but μMT and IgMi lungs comparatively had more intense inflammation visually with MD4 lungs resembling WT lungs (Figure 5A). Sample-blinded pathology scoring of WT and B cell–deficient (μMT, MD4, and IgMi) mice highlighted the increased immune cell infiltration and inflammation surrounding the blood vessels and airways of μMT and IgMi mice compared with WT and MD4 mice during F/F/S infection (Figure 5, B and C). QuPath analysis showed significantly increased immune cell density in the lungs of μMT and IgMi compared with WT mice during F/F/S infection (Figure 5D). Additionally, the frequency of inflammation and consolidation of the lungs was significantly increased in all 3 B cell–deficient strains (μMT, MD4, and IgMi) compared with WT during F/F/S infection (Figure 5E). Using BALF, we observed significantly increased lung leak in B cell–deficient mice (μMT, MD4, and IgMi) compared with WT during F/F/S infection (Figure 5F). These data indicate that loss of influenza virus–specific antibody production from B cells leads to increased lung tissue injury during F/F/S infection.

### Depletion of B cells increases formation of cytotoxic, effector memory CD8^+^ T cells in the lung.

**Figure 6:**
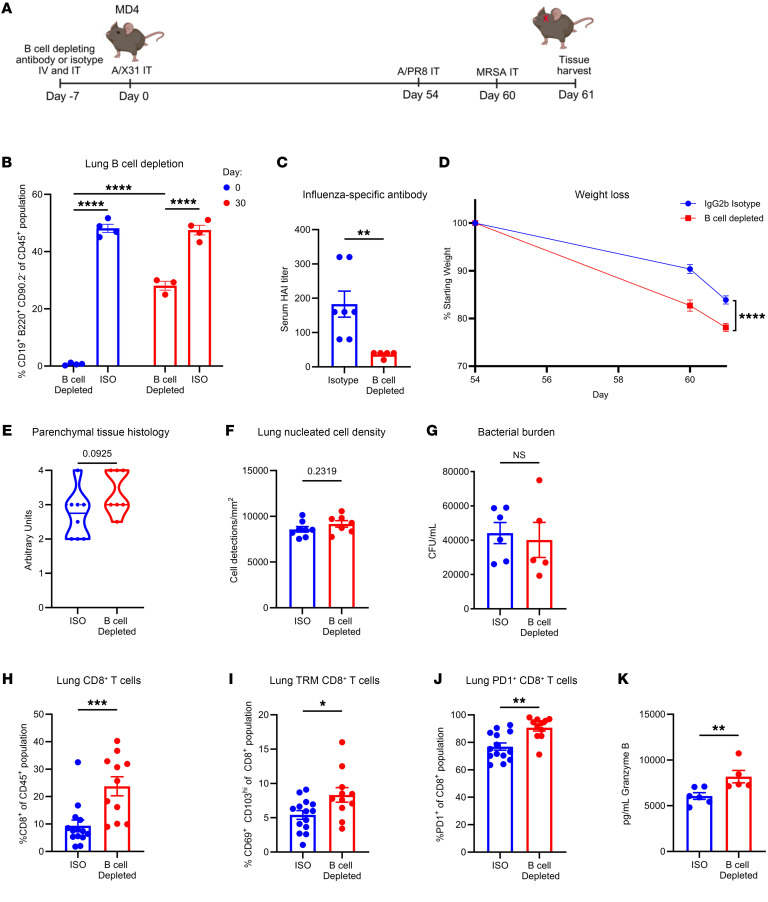
Temporal B cell depletion during primary influenza virus infection leads to increased lung memory CD8^+^ T cell formation. (**A**) B cell depletion scheme. B cells repopulated the lung before challenge with PR8 followed by MRSA. IT, intratracheal. (**B**) Depletion efficiency in the lung was confirmed via flow cytometry (day 0, isotype control [ISO]: *n* = 4; day 0, B cell–depleted: *n* = 4; day 30, ISO: *n* = 4; day 30, B cell–depleted: *n* = 3). (**C**) X-31 influenza virus–specific mouse serum titers measured by hemagglutinin inhibition assay (HAI) (ISO: *n* = 7; B cell–depleted: *n* = 5). (**D**) Percentage of weight loss for each group (ISO: *n* = 14; B cell–depleted: *n* = 12). (**E** and **F**) Histology scores of parenchymal lung sections and quantification of lung nucleated cell density (cells/mm^2^) (ISO: *n* = 8; B cell–depleted: *n* = 7). (**G**) MRSA colonies from lung homogenates (ISO: *n* = 6; B cell–depleted: *n* = 5). (**H**–**J**) Percentage of lung CD8^+^ cells, CD8^+^CD69^+^CD103^hi^ cells, and PD-1^+^CD8^+^ cells (ISO: *n* = 14; B cell–depleted: *n* = 11). (**K**) BALF granzyme B protein concentration (ISO: *n* = 6; B cell–depleted: *n* = 5). Data are represented as mean ± SEM, and *P* values were determined by repeated 2-tailed Mann-Whitney *U* test (*P < 0.05, **P < 0.01, ***P <0.001, ****P < 0.0001).

Next, we wanted to examine whether the cytotoxic, effector memory CD8^+^ T cells in the lung resulted from loss of influenza virus–specific antibody during primary influenza (X-31) virus infection or from loss of influenza virus–specific antibody during the entirety of our F/F/S model. To accomplish this, we used MD4 mice, which already have an attenuated influenza virus–specific B cell and antibody response. We depleted B cells in the lungs before primary influenza virus (X-31) infection and allowed B cells to repopulate the lung before secondary influenza virus infection (Figure 6A). Using flow cytometry, we confirmed depletion of B cells in the lung on the day of primary influenza virus challenge using a B cell–depleting antibody or isotype control (IgG2b). We observed that the B cells began to repopulate the lung as soon as 30 days after primary influenza virus infection (Figure 6B). To confirm loss of antibody during primary influenza virus infection, we assessed the serum titer of X-31–specific antibody on the day of harvesting and saw a significant decrease in B cell–depleted mice compared with the isotype controls (Figure 6C). We observed that mice with B cell depletion lost more weight during F/F/S infection compared with the isotype group, further demonstrating that weight loss during influenza virus infection was antibody dependent (Figure 6D). Although not to a statistically significant degree, we observed moderately increased lung tissue injury in the B cell–depleted group during F/F/S infection (Figure 6, E and F). Consistent with previous observations, B cell loss during primary infection did not impact MRSA burden in the lung during F/F/S infection (Figure 6G). We looked at lung CD8^+^ T cells and found that the B cell–depleted group had a significantly larger proportion of CD8^+^ T cells in the lung compared with the isotype group (Figure 6H). Additionally, there was a significant increase of CD8^+^ lung TRMs and PD-1^+^ CD8^+^ T cells in the B cell–depleted group compared with the isotype group (Figure 6, I and J). Depletion of B cells also increased the concentration of granzyme B in the BALF in comparison with the isotype controls (Figure 6K). Overall, these data show that loss of influenza virus antibody generated in response to primary challenge with influenza virus increases formation of TRM CD8^+^ T cells in the lung.

We next depleted B cells before secondary influenza challenge to explore whether loss of B cells in the lung impacts control of superinfection and CD8^+^ T cell phenotype (Supplemental Figure 9A). B cell depletion at this time point did not impact weight loss despite efficient B cell depletion in the lung, likely because of sustained high titers of influenza-specific antibody (Supplemental Figure 9, B–D). Interestingly, MRSA burden decreased in the lungs of B cell–depleted mice, but macrophage and neutrophil populations were unchanged between groups (Supplemental Figure 9, E–G). Additionally, total lung CD8^+^ T cells, memory CD8^+^ T cells (TRMs), and PD-1^+^ CD8^+^ T cells were not impacted by B cell depletion prior to secondary influenza challenge (Supplemental Figure 9, H–J). We also did not observe any differences in lung injury between groups (Supplemental Figure 9K). Overall, these data show that the presence of B cells in the lung at the heterotypic strain challenge time point is not crucial for control of superinfection.

### Passive antibody serum immunization improves control of superinfection in B cell–deficient mice.

**Figure 7:**
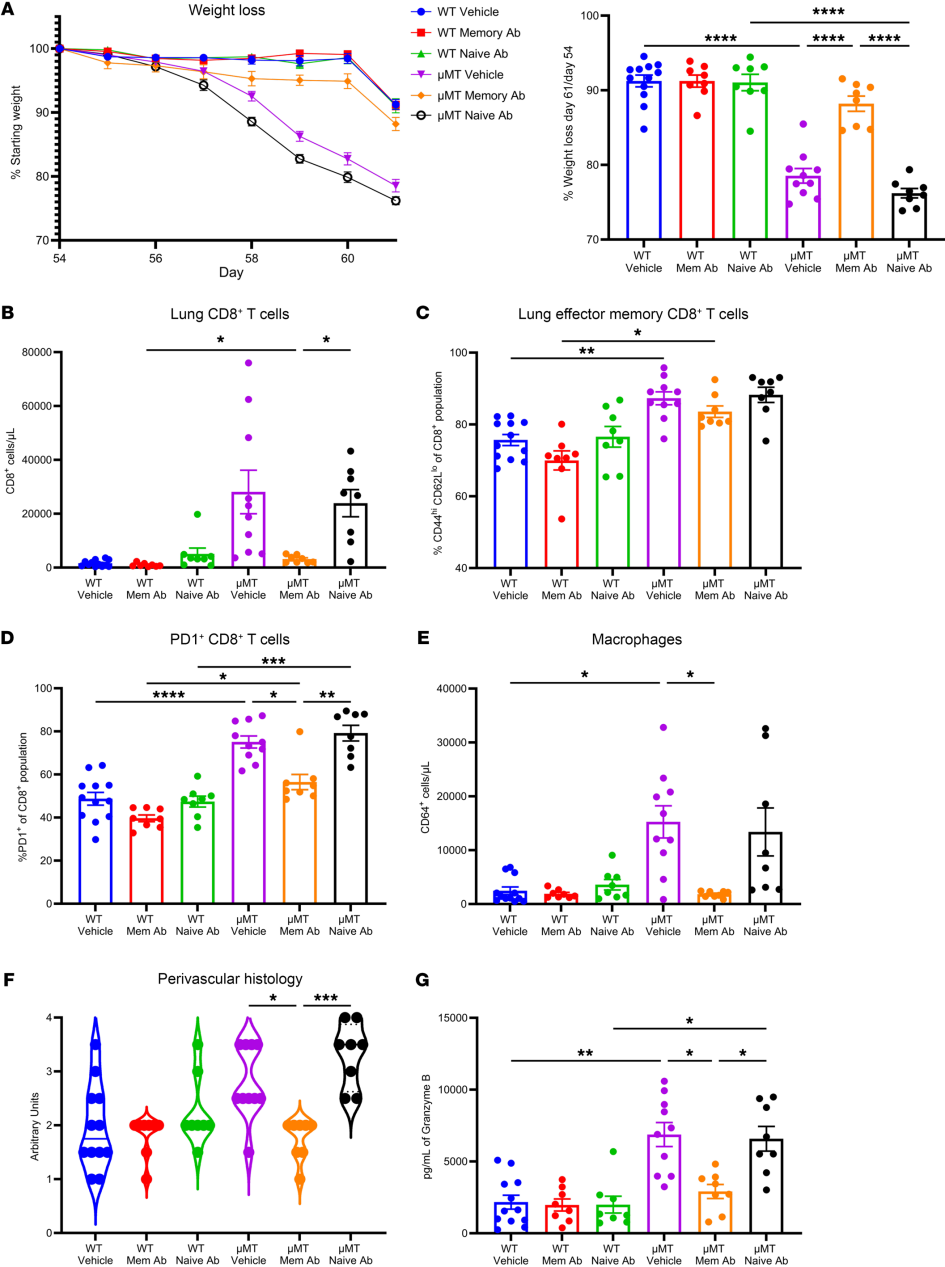
Passive immunization of μMT mice with heterotypic memory influenza serum rescues weight loss and tissue injury. WT or μMT mice were intravenously injected with 150 μL of either PBS vehicle, pooled naive WT serum, or pooled WT memory serum after PR8 challenge (day 54). (**A**) Weight loss for each group. (**B**) Flow cytometry was performed to calculate absolute number of lung CD8^+^CD45^+^CD90.2^+^ cells. (**C** and **D**) Percentage of effector memory CD44^hi^CD62^lo^CD8^+^ cells and PD-1^+^CD8^+^ cells. (**E**) Absolute number of CD64^+^CD24^–^Ly6G^–^CD45^+^CD19^–^TCRβ^–^ cells. (**F**) Histology scoring of perivascular lung sections. (**G**) BALF granzyme B protein concentration. (For **A**–**G**: WT-vehicle: *n* = 12; WT–memory Ab: *n* = 8; WT–naive Ab: *n* = 8; μMT-vehicle: *n* = 10; μMT–memory Ab: *n* = 8; μMT–naive Ab: *n* = 8). Data are represented as mean ± SEM, and *P* values were determined by repeated 1-way ANOVA measures (*P < 0.05, **P < 0.01, ***P <0.001, ****P < 0.0001).

Next, we passively immunized WT and μMT mice several hours after secondary influenza infection (PR8) with either memory serum collected and pooled from memory-superinfected WT mice, naive serum collected and pooled from WT mice, or PBS vehicle. We observed that μMT mice that received memory serum had significantly less weight loss compared with the naive serum and PBS control groups, further showing that weight loss is antibody dependent (Figure 7A). Next, we looked at viral and bacterial burden and did not observe statistically significant differences between the groups (Supplemental Figure 10, A and B). Interestingly, while we observed a decrease in total and influenza-specific lung CD8^+^ T cells in the lungs of μMT mice that received memory serum compared with controls, the proportion of CD8^+^ T cells that were effector memory did not change between groups (Figure 7, B and C, and Supplemental Figure 10C). Additionally, the proportion of CD8^+^ T cells expressing PD-1 decreased in μMT mice that received memory serum compared with controls (Figure 7D). We then examined the innate immune compartment and found that the number of macrophages decreased in the lungs of μMT mice that received memory serum, with the frequency of activated (CD40^+^) macrophages also decreased, compared with controls (Figure 7E and Supplemental Figure 10D). There was a trending decrease of NK cells and neutrophils in the lungs of μMT mice that received memory serum compared with controls, but no significant differences in number of dendritic cells (Supplemental Figure 10, E–G). We determined whether passive immunization rescued lung tissue injury, and we observed decreased lung tissue injury in μMT mice that received memory serum compared with vehicle and naive serum controls (Figure 7F and Supplemental Figure 10, H and I). Additionally, we observed a decrease in the amount of secreted granzyme B in the BALF of μMT mice that received memory serum compared with controls (Figure 7G). These data demonstrate that passive immunization with influenza-specific antibody serum rescues many of the phenotypes associated with B cell deficiency.

### B cell antibody deficiency has a similar impact on CD8^+^ T cells in influenza virus, Klebsiella pneumoniae superinfection.

After we observed a unique CD8^+^ T cell phenotype and worsened lung injury in secondary bacterial infection with MRSA in antibody-deficient mice, we examined whether our findings would be consistent with a different bacterial pathogen. To do this, we used *Klebsiella pneumoniae* (Kp), a Gram-negative bacterium that is highly virulent in mice (Supplemental Figure 11A). We observed increased weight loss in IgMi mice compared with WT mice (Supplemental Figure 11B). Unlike with F/F/S infection, bacterial burden increased in IgMi mice compared with WT (Supplemental Figure 11C). We then measured tissue integrity by BALF protein concentration and observed a significantly higher concentration in IgMi mice compared with WT (Supplemental Figure 11D). Looking at the innate immune cell compartment, we observed increased macrophages in the lungs of IgMi compared with WT mice, but not neutrophils (Supplemental Figure 11, E and F). Similar to F/F/S infection, the lungs of IgMi mice with F/F/Kp infection had higher numbers of total CD8^+^ T cells, CD8^+^ TRMs, influenza-specific CD8^+^ T cells, and CD8^+^ T cells coexpressing PD-1, LAG-3, and TIM-3 (Supplemental Figure 11, G–J). We additionally observed significantly more granzyme B secretion in the BALF of IgMi compared with WT mice (Supplemental Figure 11K). Overall, these data show that the phenotype we saw in F/F/S infection was observed in superinfections with a different pathogenic bacteria; however, IgMi mice appear to have impaired control of *K*. *pneumoniae* burden.

### Effector cytotoxic CD8^+^ T cells induced by B cell deficiency are protective against lethal secondary bacterial pneumonia.

**Figure 8:**
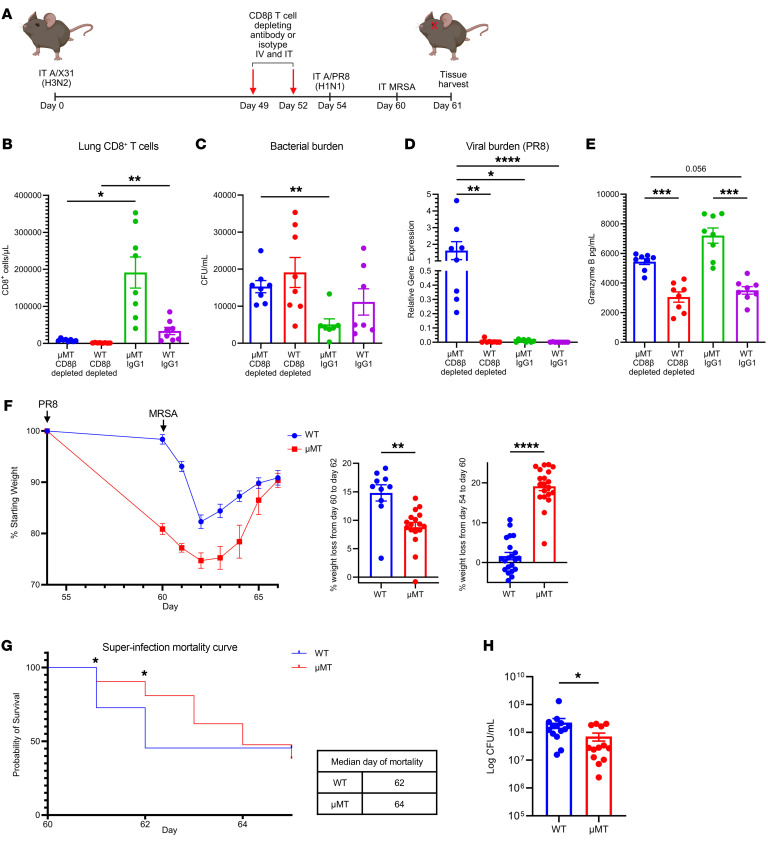
CD8^+^ T cells induced by B cell deficiency promote bacterial control during memory superinfection. (**A**) CD8^+^ T cell depletion scheme. IT, intratracheal. (**B**) CD8β T cell depletion was confirmed on day of tissue harvesting via flow cytometry (μMT–CD8β-depleted: *n* = 8; WT–CD8β-depleted: *n* = 8; μMT-ISO: *n* = 8; WT-ISO: *n* = 8). (**C**) MRSA burden in lung homogenates (μMT–CD8β-depleted: *n* = 8; WT–CD8β-depleted: *n* = 8; μMT-ISO: *n* = 7; WT-ISO: *n* = 7). (**D**) Viral protein (PR8) was assessed via quantitative PCR (μMT–CD8β-depleted: *n* = 8; WT–CD8β-depleted: *n* = 8; μMT-ISO: *n* = 8; WT-ISO: *n* = 8). (**E**) BALF granzyme B protein concentration (μMT–CD8β-depleted: *n* = 8; WT–CD8β-depleted: *n* = 8; μMT-ISO: *n* = 8; WT-ISO: *n* = 8). (**F**) WT and μMT mice were challenged with a lethal dose of MRSA (2 × 10^8^) during memory superinfection and weighed daily (left). PR8-induced weight loss prior to MRSA challenge (day 54 to day 60) (right), and percentage of weight loss 48 hours following MRSA challenge (day 62) (middle) (WT: *n* = 22; μMT: *n* = 21). (**G**) Left: Survival percentage was calculated daily after lethal MRSA challenge. Right: Median day of mortality was calculated for WT and μMT groups (WT: *n* = 22; μMT: *n* = 21). (**H**) MRSA burden 14 hours after lethal MRSA challenge in lung homogenate (WT: *n* = 14; μMT: *n* = 13). Data are represented as mean ± SEM. For **B**–**E**, *P* values were determined by repeated 1-way ANOVA measures; for **F**–**H**, *P* values were determined by repeated 2-tailed Mann-Whitney *U* test (*P < 0.05, **P < 0.01, ***P <0.001, ****P < 0.0001).

To assess whether memory effector CD8^+^ T cells in the lung were protective or detrimental during secondary bacterial infection, we depleted CD8β^+^ T cells (intravenously and intratracheally) in WT and μMT mice before challenge with influenza (PR8) virus (Figure 8A). Lung CD8β^+^ T cell depletion was confirmed on the day of tissue harvesting using flow cytometry (Figure 8B). We observed that depletion of CD8β^+^ T cells in μMT mice resulted in significantly higher MRSA burden in the lung in comparison with controls (Figure 8C). Additionally, μMT lungs depleted of CD8β^+^ T cells had significantly higher viral burden (PR8) compared with WT and isotype groups (Figure 8D). This suggests that the cytotoxic memory CD8^+^ T cells of μMT are protective against influenza virus and MRSA challenge; however, depletion of CD8^+^ T cells in WT mice did not significantly impact influenza virus or MRSA burden, likely because of the presence of antibody. Because we observed previously that B cell–deficient mice (μMT, MD4, and IgMi) formed lung CD8^+^ T cells with increased granzyme B expression, we analyzed the concentration of granzyme B in the BALF. We observed that μMT mice with CD8β T cell depletion had moderately decreased granzyme B concentration in the BALF, although this did not reach statistical significance. There was no difference in granzyme B concentration in the BALF of WT groups (CD8^+^ T cell depleted vs. isotype), demonstrating that effector cytotoxic lung CD8^+^ T cell formation is a unique feature of B cell deficiency (Figure 8E). We did not observe any differences in lung tissue injury between CD8β^+^ T cell–depleted and isotype groups (Supplemental Figure 12A). However, we saw a significant increase of lung CD4^+^ T cell number, IL-6, and BALF neutrophil frequency in μMT mice that were depleted of CD8β^+^ T cells, suggesting that other immune cells were recruited to the lungs to control superinfection (Supplemental Figure 12, B–D).

Since we saw that CD8^+^ T cells are important for MRSA clearance in the lungs of μMT mice, we wanted to assess their ability to control secondary bacterial pneumonia by challenging WT and μMT mice with a lethal dose (2 × 10^8^ CFU) of MRSA in our model. We observed that μMT mice lost more weight than WT mice (days 54–60), which was dependent on influenza virus (PR8) infection. WT mice lost a significant amount of weight in the 48 hours following MRSA challenge (days 60–62), suggesting that weight loss in WT mice is primarily dependent on secondary bacterial infection (Figure 8F). Overall, WT and μMT groups had similar mortality rates following lethal superinfection, but the time points at which mortality occurred significantly differed. Mortality in the WT group occurred 24–48 hours after MRSA challenge, suggesting that WT mice were not able to effectively clear MRSA infection (Figure 8G). The μMT group experienced less mortality 24–48 hours after MRSA challenge (20%) compared with WT mice (65%) (Figure 8G). These data suggest that the μMT mice were able to control the MRSA infection more effectively than the WT group despite already experiencing significant weight loss due to influenza virus infection. Forty-eight hours after MRSA challenge, both μMT and WT groups began to recover from the weight loss; however, some individual μMT mice continued to lose weight and were euthanized at days 63, 64, and 65 (Figure 8, F and G). The continued mortality 72–96 hours after MRSA challenge in the μMT group was likely due to antibody-dependent weight loss and the inflammatory lung environment. To determine whether the increased early mortality in WT mice was due to increased MRSA burden, we examined bacterial burden 14 hours after lethal MRSA challenge and observed increased burden in WT compared with μMT mice (Figure 8H). When heterotypic memory–experienced WT and μMT mice were challenged with lethal MRSA without secondary PR8 influenza virus challenge (Supplemental Figure 12E), both groups had about 50% mortality within 48 hours of challenge (Supplemental Figure 12, F and G). Overall, these data suggest that loss of B cells induces formation of lung cytotoxic effector CD8^+^ T cells, which promote acute control of MRSA challenge during F/F/S infection.

## Discussion

Pulmonary infections caused by viruses, such as influenza virus and SARS-CoV-2, remain a persistent public health burden globally. Immune dysregulation following viral infection increases susceptibility to developing secondary bacterial pneumonias, resulting in increased morbidity and mortality rates (5, 7, 8). Studies on naive mice have shown that altered immune responses during preceding viral infection hinder antibacterial mechanisms during secondary bacterial pneumonia (9–16). Our group has previously shown that mice with heterotypic influenza memory have better-controlled immunopathology and improved bacterial clearance in the lung (32). The data presented in this study expand on this knowledge and show that previous influenza virus infection alters immune responses against secondary bacterial infection. CD8^+^ T cells are crucial for controlling heterotypic influenza infection by recognizing and killing virally infected cells, but less is known about their responses to extracellular bacterial infections. While primarily an extracellular infection, MRSA can invade phagocytic cells. CD8^+^ T cells can lyse and kill these infected cells via perforin and granzyme B (41, 42). Our analysis of sorted lung CD8^+^ T cells in WT mice showed transcriptomic differences between F/F and F/F/S groups. Th17-derived cytokines and antimicrobial peptides are important for clearance of bacterial infection, and induction of type I IFNs by influenza virus A has been shown to prevent Th17 activation (10, 11, 13). Our results suggest that MRSA challenge downregulates proinflammatory pathways and genes associated with antibacterial clearance, such as IL-17a, IL-23a, LCN2, IL-1α, IL-1β, and S100A8–9, consistent with previous findings (11). During influenza virus infection, the proinflammatory cytokines IFN-γ and TNF-α are upregulated, but we found that expression of these cytokines by lung CD8^+^ T cells decreased significantly as a result of MRSA infection in WT mice. IFN-γ has been shown previously to impair control of secondary bacterial pneumonias, so downregulation of IFN-γ in our model may be beneficial (13, 43, 44). However, downregulation of TNF-α may be detrimental for bacterial clearance as it has been shown to be protective against *S*. *aureus* (45–47). Together, these data show that despite memory training of immune cells, secondary challenge with MRSA alters CD8^+^ T cell phenotype; however, these cells appear to be able to effectively mount an antibacterial response against MRSA. Further investigation is needed to uncover phenotypic and functional changes in CD8^+^ T cells during superinfection.

B cells have been shown to be integral to maintaining tissue integrity, primarily by immunoregulating key effector cells that drive tissue damage, neutrophils and macrophages (48–50). We observed that loss of B cells increased lung injury and inflammation, but this finding was seen only after heterotypic memory, not acute superinfection. Increased inflammation and immune cell recruitment in B cell–deficient lungs were particularly concentrated toward the blood vessels, rather than the parenchyma. A similar finding has been seen in lungs of Rag2^–/–^ mice, which lack mature B and T cells (51). We examined whether increased lung tissue injury was due to lack of antibody or due to other antibody-independent B cell functions (48–50). Using MD4 and IgMi mice in addition to μMT mice showed that lung tissue injury during memory superinfection was caused primarily by loss of antibody. IgMi mice have been reported previously to secrete more IL-10, an important immunoregulatory cytokine, but increased B cell–derived IL-10 did not rescue lung tissue injury (40, 52). Depleting CD8^+^ T cells did not rescue lung tissue injury. There was an increase in the lungs of B cell–deficient mice of cytokines that are associated with immunopathology, particularly IL-6, MCP-1, MIP-1β, and G-CSF (53–56). Cytokine increases suggest that absence of B cell immunoregulatory signals to neutrophils and macrophages may cause increased lung tissue inflammation during memory superinfection. Additionally, we saw increased inflammatory macrophages in B cell–deficient mice compared with WT mice. While there was no difference in total neutrophil number between WT and B cell–deficient lungs, the neutrophils of B cell–deficient mice appeared to be more mature and had increased iNOS expression, which was shown to increase tissue damage (57–59). Add-back of influenza memory antibody serum to B cell–deficient mice at the beginning of superinfection rescued weight loss, lung injury, and decreased numbers of activated CD8^+^ T cells and macrophages. Overall, these findings show that loss of antibody production by B cells leads to a more inflammatory environment, leading to increased lung injury. However, the immunological mechanisms underlying this increased lung injury require further investigation.

Clinically, depletion of B cells via monoclonal antibodies like rituximab increases patient risk of higher morbidity and mortality from infectious diseases (60). Previous studies have shown that loss of B cells accelerates the contraction of memory CD8^+^ T cells formed in response to infection and vaccination (61–66). Rituximab-treated rheumatoid arthritis patients were shown to have impaired expansion of influenza virus–specific CD8^+^ T cells following influenza vaccination, a finding also seen in B cell–deficient mice challenged with influenza virus (62). However, a different study examining responses to COVID-19 mRNA vaccines in rituximab-treated patients showed that the expansion of antigen-specific CD8^+^ T cells was not impaired, but rather the CD8^+^ T cells were more activated and expressed more effector molecules (63). We observed that expansion of effector and resident memory CD8^+^ T cells in B cell–deficient mice was not impaired. The number of lung CD8^+^ T cells and influenza virus–specific CD8^+^ T cells was significantly higher in B cell–deficient mice compared with WT. Additionally, we saw an increased population of CD8^+^ TRMs in the lungs of B cell–deficient mice compared with WT. We did not observe impairment in the formation of memory CD8^+^ T cells in B cell–deficient models during heterotypic memory superinfection. The difference in findings is possibly due to natural infection rather than vaccination for the primary challenge.

We observed that CD8^+^ T cells in B cell–deficient mice expressed more T cell regulatory markers (PD-1, LAG-3, TIM-3) than WT CD8^+^ T cells, with expression further increased after MRSA challenge. CD8^+^ T cells in B cell–deficient mice had a diminished antiviral phenotype, expressing lower levels of IFN-γ and TNF-α compared with WT CD8^+^ T cells. However, the CD8^+^ T cells expressed significantly more granzyme B compared with WT, a finding that is consistent with a previous study showing impaired CD8^+^ T cell responses in MD4 mice (61). We also found that genes commonly associated with T cell regulation and exhaustion were increased in CD8^+^ T cells from B cell–deficient mice compared with WT. This suggests that B cell deficiency may be driving CD8^+^ T cells toward a state of terminal effector differentiation. It is clear that B cells are critically involved in regulating memory CD8^+^ T cell responses, but it is unclear what mechanism(s) are responsible. We found that loss of antibody secretion by B cells was responsible for the increase in lung effector CD8^+^ T cells, as the CD8^+^ T cells observed in IgMi mice were very similar to those seen in μMT mice. B cells from IgMi mice have been previously shown to have a higher propensity to secrete IL-10 and participate in germinal center reactions compared with those from WT mice (40). We saw that IgMi mice had a higher proportion of effector memory CD8^+^ T cells compared with μMT mice, which may be due to increased germinal center interactions. Depletion of B cells in MD4 mice and adding back of influenza antibody serum showed that loss of antibody results in increased memory CD8^+^ T cells during secondary challenge. Studies are needed to show mechanistically how loss of antibodies during primary infection impacts CD8^+^ T cell responses. We theorize that lack of antibody results in prolonged engagement of antigen with T cell receptors on CD8^+^ T cells, which has been shown to increase the magnitude of cytotoxic T lymphocyte activity in CD8^+^ T cells (67–69).

Heterotypic influenza memory resulted in lower MRSA burden in the lung during superinfection in comparison with previously naive mice (32), so it was interesting that loss of memory B cells did not impact MRSA burden. However, secondary challenge with *Klebsiella pneumoniae* did result in higher bacterial burdens in IgMi mice compared with WT, which could be due to increased virulence or increased ability to evade host immune responses (70). Cytotoxic CD8^+^ T cells are important mediators of intracellular pathogen killing through production of cytokines, such as IFN-γ, TNF-α, and granzyme B, and killing of infected cells (71). While only a subset of the CD8^+^ T cells (15%–50%) in our B cell–deficient mice were influenza virus specific, a majority of them expressed granzyme B. This suggests bystander activation of CD8^+^ T cells, which produce high levels of granzyme B and perforin (72). Depleting CD8β^+^ T cells before superinfection increased viral and MRSA burden only in μMT lungs, demonstrating their necessity for viral and bacterial clearance. Challenging WT and μMT mice with lethal MRSA infection during superinfection resulted in death of WT mice within 24–48 hours of infection, which was likely due to higher MRSA burden in the lung. However, μMT mice largely survived initial bacterial infection but died at later time points, likely as a result of increased weight loss and lung inflammation. The delayed mortality seen in μMT mice was only observed when the mice were challenged with PR8 before lethal MRSA infection, suggesting that PR8 infection activated bystander CD8^+^ T cells, which promoted clearance of secondary bacterial infection. These data show that the cytotoxic effector memory CD8^+^ T cells that result from B cell deficiency are protective against secondary bacterial challenge, but their contributions toward immunopathology are still unclear. These data suggest that the increased lung tissue injury and inflammation seen in B cell–deficient mice during memory superinfection are likely due to immune activation from bacterial challenge rather than bacterial load. Although our findings are limited to two heterotypic influenza virus strains, we believe the immunological findings regarding regulation of CD8^+^ T cells by B cells are relevant to other antimicrobial immunity and vaccination contexts. Further studies are needed to resolve how B cells and CD8^+^ T cells regulate each other, which is crucial for providing effective therapeutic interventions in viral and bacterial infections.

## Methods

### Sex as a biological variable.

This study examined predominantly male animals to reduce experimental variability. Some experiments (as indicated) were done with female mice, and similar findings are reported for both sexes.

### Mice.

Six- to eight-week-old male WT C57BL/6, MD4 [C57BL/6-Tg(IghelMD4)4Ccg/J], and μMT (B6.129S2-Ighmtm1Cgn/J) mice were purchased from The Jackson Laboratory. IgMi mice were a gift from the laboratory of Timothy Hand at UPMC Children’s Hospital of Pittsburgh. Mice were maintained under pathogen-free conditions at UPMC Children’s Hospital of Pittsburgh. Studies were performed on sex- and age-matched mice.

### Mouse model and sample collection.

On day 0, 6- to 8-week-old male or female mice were infected with 0.5 × 10^5^ to 1 × 10^5^ PFU of mouse-adapted influenza virus A/HKx31 H3N2 (X-31) or PBS vehicle. After 54 days, the mice were rechallenged with 10^3^ PFU of a heterotypic strain of mouse-adapted influenza virus A/PR/8/34 H1N1 (PR8) or PBS vehicle. Six days after influenza virus rechallenge (day 60), the mice were challenged with 5 × 10^7^ CFU of USA300 MRSA (stationary phase) suspended in PBS or vehicle, and tissues were harvested a day later. For homotypic influenza virus challenge models, mice were infected with PR8 at both viral challenge time points. For *Klebsiella pneumoniae* experiments, mice were challenged with 5 × 10^3^ CFU of *K*. *pneumoniae* (ATCC 43816) at day 60, and tissues were harvested 48 hours later (day 62). All infections were given via oropharyngeal instillation. Mice were euthanized via pentobarbital injection followed by cervical dislocation and exsanguination by severing of the renal artery.

### BALF collection and differential cell staining.

Bronchoalveolar lavage fluid (BALF) was collected from mice at tissue harvesting and processed as described in Supplemental Methods.

### Bacterial plating.

The upper right lung lobes of mice were collected and homogenized in 1 mL of PBS. Neat and 10-fold dilutions of lung homogenate were dot-plated and then incubated at 37°C overnight, and CFUs were quantified by bacterial colony counting.

### Flow cytometry.

Flow cytometry was performed on single-cell suspensions obtained from lung tissue as described in Supplemental Methods. Antibodies utilized in the study are listed in Supplemental Table 1.

### Histology and QuPath analysis.

Histological processing and analysis of lungs were performed as outlined in Supplemental Methods.

### RNA extraction and quantitative PCR.

Lung tissue was collected at day of harvesting and processed for RNA extraction and quantitative PCR as described in Supplemental Methods.

### Hemagglutinin inhibition assay.

Hemagglutinin inhibition assays were conducted on serum samples as described in Supplemental Methods.

### Bio-Plex and protein assays.

Protein levels in BALF were determined using the Pierce BCA protein assay kit (Thermo Fisher Scientific). Cytokine production was measured using homogenate of upper right lung (1 mL of PBS) with the Bio-Rad Magpix platform with the Bio-Plex Pro Mouse Cytokine 23-plex assay (Bio-Rad). BALF was used to determine granzyme B production with a mouse granzyme B DuoSet ELISA kit (R&D Systems).

### CD8β T cell depletion.

CD8β T cell depletion was performed twice, 5 and 2 days before PR8 challenge (days 49 and 52). μMT or WT C57BL/6 mice were injected intravenously via tail vein with 200 μg of *InVivo*MAb anti–mouse CD8β (Lyt 3.2, clone 53-5.8, Bio X Cell) or 200 μg of *InVivo*MAb rat IgG1 isotype control, anti–horseradish peroxidase (clone HPRN, Bio X Cell). Mice were oropharyngeally given 200 μg of *InVivo*MAb anti–mouse CD8β (Lyt 3.2, clone 53-5.8, Bio X Cell) or 200 μg of *InVivo*MAb rat IgG1 isotype control, anti–horseradish peroxidase (clone HPRN, Bio X Cell). Depletion efficiency was assessed via flow cytometry.

### B cell depletion.

Early B cell depletion was performed 7 days before X-31 (H3N2) challenge. MD4 mice were injected intravenously via tail vein with 250 μg of Ultra-Leaf Purified anti–mouse CD20 antibody (SA271G2, BioLegend) or with 250 μg of rat IgG2b κ isotype control (RTK4530, BioLegend). Mice were oropharyngeally given 125 μg of Ultra-Leaf Purified anti–mouse CD20 antibody (SA271G2, BioLegend) or 125 μg of rat IgG2b κ isotype control (RTK4530, BioLegend). Late B cell depletion was performed intravenously and oropharyngeally with identical CD20 antibody and isotype volume and concentrations except that treatment was performed 5 days (day 49) and 2 days (day 52) before PR8 infection (day 54) in WT (C57BL/6) mice.

### Serum transfer experiments.

WT or μMT mice were injected intravenously via tail vein with 150 μL of PBS vehicle, pooled naive serum collected from WT mice, or pooled influenza memory serum collected from WT mice with F/F/S infection at day of tissue harvesting (day 61). Mice were passively immunized 6–7 hours after PR8 challenge (day 54).

### Bulk RNA sequencing on lung CD8^+^ T cells.

RNA was extracted from mouse lung CD8^+^ T cells according to methods outlined in Supplemental Methods. MedGenome performed library preparation (Takara SMART-Seq mRNA) and sequencing (Illumina NovaSeq) with 100-bp single-end reads and 20 million reads per sample.

### Bioinformatics.

Bulk RNA sequencing reads were aligned and analyzed by MedGenome. Gene set enrichment analysis was performed using WebGestalt (WEB-based GEne SeT AnaLysis Toolkit) (73). Heatmaps were made using the provided transcripts per million (TPM) counts and using R in R Studio version 4.1.0. Data were log_2_-transformed and scaled according to row using the pheatmap package (74) to highlight expression levels for select genes across samples.

### Statistics.

Data were analyzed using GraphPad Prism software. Experiments were repeated 2–6 times as indicated. All data are presented as mean with SEM, unless otherwise noted. Mann-Whitney *U* test, 1-way ANOVA with multiple comparisons, or 2-way ANOVA was used for statistical significance with a *P* value less than or equal to 0.05.

### Study approval.

All research with animal models was subject to prior review and approval by the University of Pittsburgh’s Institutional Animal Care and Use Committee (protocol 23073501) and was conducted in compliance its guidelines.

### Data availability.

Datasets are available in the NCBI’s Gene Expression Omnibus (GEO) repository (GSE288913). Individual data points are graphed to depict experimental variation. Raw data are provided in the Supporting Data Values file. Data are available upon request, subject to institutional review and approval.

## Author contributions

LMM and JFA conceptualized the study. LMM, EMC, AMD, FR, BPD, KLP, and AG developed methodology. LMM analyzed data and wrote the original draft. LMM and JFA reviewed and edited the manuscript. JFA supervised the study.

## Supplementary Material

Supplemental data

Supporting data values

## Version 1 06/10/2025

In-Press Preview

## Version 2 08/15/2025

Electronic publication

## References

[1] Matrosovich MN (2004). Human and avian influenza viruses target different cell types in cultures of human airway epithelium. Proc Natl Acad Sci U S A.

[2] Atkin-Smith GK (2018). The induction and consequences of influenza A virus-induced cell death. Cell Death Dis.

[3] Robinson KM (2015). The immunology of influenza virus-associated bacterial pneumonia. Curr Opin Immunol.

[4] Cipolla EM (2020). Influenza sequelae: from immune modulation to persistent alveolitis. Clin Sci (Lond).

[5] Louria DB (1959). Studies on influenza in the pandemic of 1957-1958. II. Pulmonary complications of influenza. J Clin Invest.

[6] Van de Sandt CE (2017). Human CD8^+^ T cells damage noninfected epithelial cells during influenza virus infection in vitro. Am J Respir Cell Mol Biol.

[7] Morens DM (2008). Predominant role of bacterial pneumonia as a cause of death in pandemic influenza: implications for pandemic influenza preparedness. J Infect Dis.

[8] Kuiken T, Taubenberger JK (2008). Pathology of human influenza revisited. Vaccine.

[9] Robinson KM (2013). Influenza A exacerbates Staphylococcus aureus pneumonia by attenuating IL-1β production in mice. J Immunol.

[10] Nakamura S (2011). Synergistic stimulation of type I interferons during influenza virus coinfection promotes Streptococcus pneumoniae colonization in mice. J Clin Invest.

[11] Kudva A (2011). Influenza A inhibits Th17-mediated host defense against bacterial pneumonia in mice. J Immunol.

[12] Ghoneim HE (2013). Depletion of alveolar macrophages during influenza infection facilitates bacterial superinfections. J Immunol.

[13] Sun K, Metzger DW (2008). Inhibition of pulmonary antibacterial defense by interferon-gamma during recovery from influenza infection. Nat Med.

[14] Small CL (2010). Influenza infection leads to increased susceptibility to subsequent bacterial superinfection by impairing NK cell responses in the lung. J Immunol.

[15] Narayana Moorthy A (2013). In vivo and in vitro studies on the roles of neutrophil extracellular traps during secondary pneumococcal pneumonia after primary pulmonary influenza infection. Front Immunol.

[16] Rich HE (2019). Interferon lambda inhibits bacterial uptake during influenza superinfection. Infect Immun.

[17] Wasserman MG (2022). Airway bacterial colonization, biofilms and blooms, and acute respiratory infection. Pediatr Crit Care Med.

[18] Li N (2015). Influenza viral neuraminidase primes bacterial coinfection through TGF-β-mediated expression of host cell receptors. Proc Natl Acad Sci U S A.

[19] Gomez Lorenzo MM, Fenton MJ (2013). Immunobiology of influenza vaccines. Chest.

[20] Krammer F (2019). The human antibody response to influenza A virus infection and vaccination. Nat Rev Immunol.

[21] Doherty PC (2006). Influenza and the challenge for immunology. Nat Immunol.

[22] Pizzolla A, Wakim LM (2019). Memory T cell dynamics in the lung during influenza virus infection. J Immunol.

[23] Zens KD (2016). Vaccine-generated lung tissue-resident memory T cells provide heterosubtypic protection to influenza infection. JCI Insight.

[24] Pizzolla A (2017). Resident memory CD8^+^ T cells in the upper respiratory tract prevent pulmonary influenza virus infection. Sci Immunol.

[25] Shenoy AT (2020). Lung CD4^+^ resident memory T cells remodel epithelial responses to accelerate neutrophil recruitment during pneumonia. Mucosal Immunol.

[26] van de Wall S (2021). Influenza-specific lung-resident memory CD8^+^ T cells. Cold Spring Harb Perspect Biol.

[27] Lee S (2024). Tissue-resident memory T cells in protective immunity to influenza virus. Curr Opin Virol.

[28] Connors TJ (2016). Airway CD8(+) T cells are associated with lung injury during infant viral respiratory tract infection. Am J Respir Cell Mol Biol.

[29] Chowdhury D, Lieberman J (2008). Death by a thousand cuts: granzyme pathways of programmed cell death. Annu Rev Immunol.

[30] Alexandrova Y (2024). Dynamics of pulmonary mucosal cytotoxic CD8 T-cells in people living with HIV under suppressive antiretroviral therapy. Respir Res.

[31] Price O (2022). Epidemiology of repeat influenza infection in Queensland, Australia, 2005–2017. Epidemiol Infect.

[32] Cipolla EM (2022). Heterotypic influenza infections mitigate susceptibility to secondary bacterial infection. J Immunol.

[33] Rutigliano JA (2010). Protective memory responses are modulated by priming events prior to challenge. J Virol.

[34] Kreijtz JH (2007). Primary influenza A virus infection induces cross-protective immunity against a lethal infection with a heterosubtypic virus strain in mice. Vaccine.

[35] Li H (2019). Dysfunctional CD8 T cells form a proliferative, dynamically regulated compartment within human melanoma. Cell.

[36] van der Leun AM (2020). CD8^+^ T cell states in human cancer: insights from single-cell analysis. Nat Rev Cancer.

[37] Rha MS, Shin EC (2021). Activation or exhaustion of CD8^+^ T cells in patients with COVID-19. Cell Mol Immunol.

[38] Jenkins E (2023). The current state and future of T-cell exhaustion research. Oxf Open Immunol.

[39] Mozdzanowska K (2005). Roles of CD4+ T-cell-independent and -dependent antibody responses in the control of influenza virus infection: evidence for noncognate CD4+ T-cell activities that enhance the therapeutic activity of antiviral antibodies. J Virol.

[40] Sahputra R (2018). Evaluating the IgMi mouse as a novel tool to study B-cell biology. Eur J Immunol.

[41] Shepherd FR, McLaren JE (2020). T cell immunity to bacterial pathogens: mechanisms of immune control and bacterial evasion. Int J Mol Sci.

[42] Bröker BM (2016). The T cell response to Staphylococcus aureus. Pathogens.

[43] Greenlee-Wacker MC, Nauseef WM (2017). IFN-γ targets macrophage-mediated immune responses toward *Staphylococcus aureus*. J Leukoc Biol.

[44] Verma AK (2021). IFN-γ drives TNF-α hyperproduction and lethal lung inflammation during antibiotic treatment of postinfluenza Staphylococcus aureus pneumonia. J Immunol.

[45] Damjanovic D (2011). Negative regulation of lung inflammation and immunopathology by TNF-α during acute influenza infection. Am J Pathol.

[46] Varley CD (2013). Persistence of Staphylococcus aureus colonization among individuals with immune-mediated inflammatory diseases treated with TNF-α inhibitor therapy. Rheumatology (Oxford).

[47] Giai C (2013). Shedding of tumor necrosis factor receptor 1 induced by protein A decreases tumor necrosis factor alpha availability and inflammation during systemic Staphylococcus aureus infection. Infect Immun.

[48] Suchanek O, Clatworthy MR (2023). Homeostatic role of B-1 cells in tissue immunity. Front Immunol.

[49] Gawish R (2022). A neutrophil-B-cell axis impacts tissue damage control in a mouse model of intraabdominal bacterial infection via Cxcr4. Elife.

[50] Suchanek O (2023). Tissue-resident B cells orchestrate macrophage polarisation and function. Nat Commun.

[51] Teijaro JR (2010). Memory CD4 T cells direct protective responses to influenza virus in the lungs through helper-independent mechanisms. J Virol.

[52] Sun Z (2023). B cell-derived IL-10 promotes the resolution of lipopolysaccharide-induced acute lung injury. Cell Death Dis.

[53] Liu Q (2016). The cytokine storm of severe influenza and development of immunomodulatory therapy. Cell Mol Immunol.

[54] Coates BM (2018). Inflammatory monocytes drive influenza A virus-mediated lung injury in juvenile mice. J Immunol.

[55] Hall MW (2013). Innate immune function and mortality in critically ill children with influenza: a multicenter study. Crit Care Med.

[56] Tanaka T (2014). IL-6 in inflammation, immunity, and disease. Cold Spring Harb Perspect Biol.

[57] Deniset JF (2017). Splenic Ly6G^high^ mature and Ly6G^int^ immature neutrophils contribute to eradication of S. pneumoniae. J Exp Med.

[58] Pfirschke C (2020). Tumor-promoting Ly-6G^+^ SiglecF^high^ cells are mature and long-lived neutrophils. Cell Rep.

[59] Saini R, Singh S (2018). Inducible nitric oxide synthase: an asset to neutrophils. J Leukoc Biol.

[60] Varley CD, Winthrop KL (2021). Long-term safety of rituximab (risks of viral and opportunistic infections). Curr Rheumatol Rep.

[61] Klarquist J (2021). B cells promote CD8 T cell primary and memory responses to subunit vaccines. Cell Rep.

[62] Graalmann T (2021). B cell depletion impairs vaccination-induced CD8^+^ T cell responses in a type I interferon-dependent manner. Ann Rheum Dis.

[63] Madelon N (2022). Robust T-cell responses in anti-CD20-treated patients following COVID-19 vaccination: a prospective cohort study. Clin Infect Dis.

[64] Van Meerhaeghe T (2023). Regulation of CD8 T cell by B-cells: a narrative review. Front Immunol.

[65] Shen H (2003). A specific role for B cells in the generation of CD8 T cell memory by recombinant Listeria monocytogenes. J Immunol.

[66] Asano MS, Ahmed R (1996). CD8 T cell memory in B cell-deficient mice. J Exp Med.

[67] Prlic M (2006). Duration of the initial TCR stimulus controls the magnitude but not functionality of the CD8+ T cell response. J Exp Med.

[68] Iezzi G (1998). The duration of antigenic stimulation determines the fate of naive and effector T cells. Immunity.

[69] Abdelbary M (2023). T cell receptor signaling strength establishes the chemotactic properties of effector CD8^+^ T cells that control tissue-residency. Nat Commun.

[70] Sá-Pessoa J (2025). Klebsiella pneumoniae emerging anti-immunology paradigms: from stealth to evasion. Trends Microbiol.

[71] Balin SJ (2018). Human antimicrobial cytotoxic T lymphocytes, defined by NK receptors and antimicrobial proteins, kill intracellular bacteria. Sci Immunol.

[72] Kim TS, Shin EC (2019). The activation of bystander CD8^+^ T cells and their roles in viral infection. Exp Mol Med.

[73] Zhang B (2005). WebGestalt: an integrated system for exploring gene sets in various biological contexts. Nucleic Acids Res.

[74] https://cran.r-project.org/web//packages//pheatmap/pheatmap.pdf.

