# Supplementary material for: B cell deficiency induces cytotoxic memory CD8+ T cells during influenza-associated bacterial pneumonia
Source: J Clin Invest. 2025 Jun 10;135(16):e188342. doi: 10.1172/JCI188342 (PMC12352901; doi:10.1172/JCI188342)
Supplement: Supplemental data [file jci-135-188342-s225.pdf]

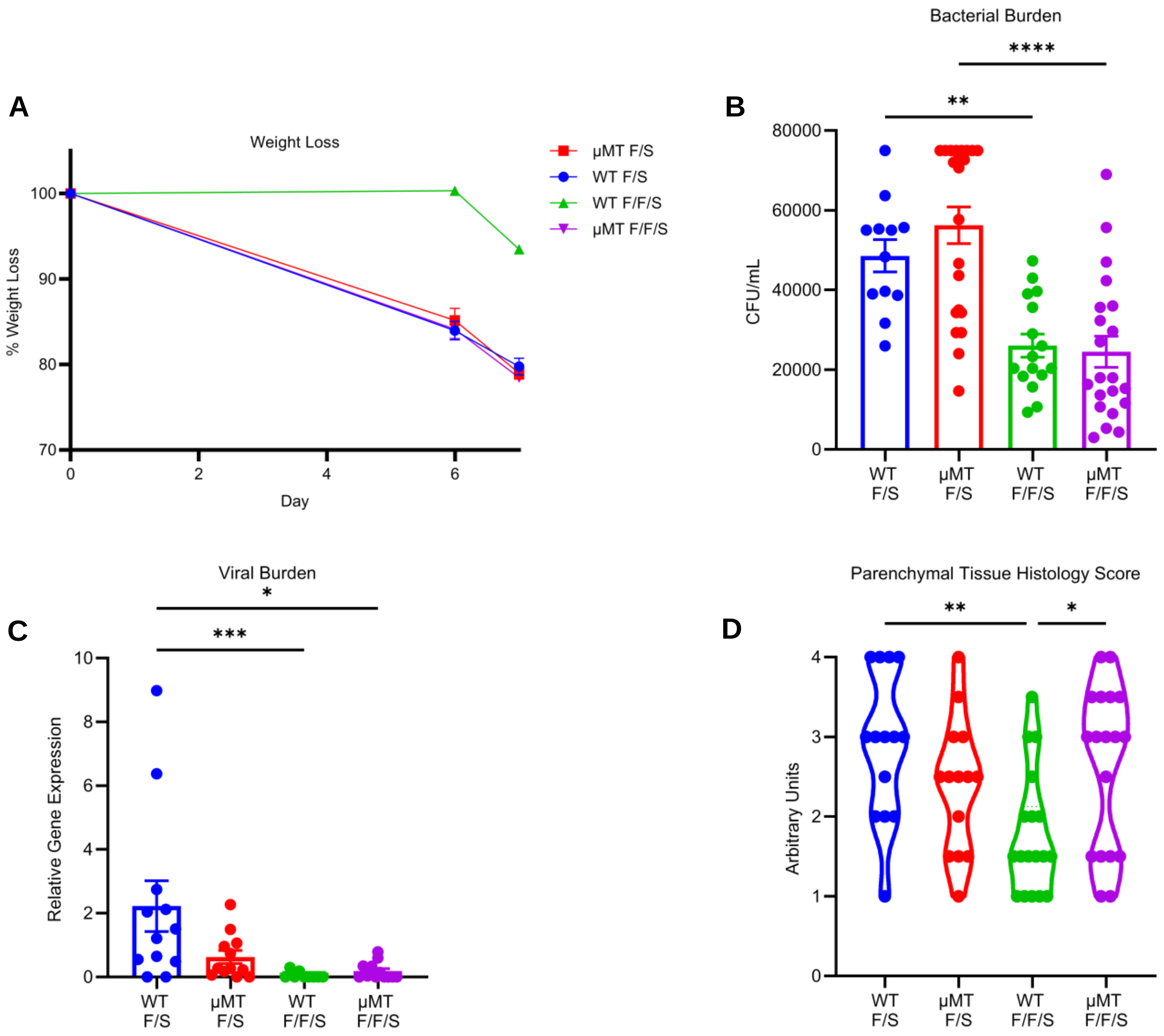

SUPPLEMENTAL FIGURE 1. WT and µMT mice have decreased MRSA and viral lung burden and weight loss with heterotypic influenza memory. **(A)** Percent of weight loss was calculated starting from secondary infection with PR8 for each treatment group (WT-F/S *n*=14, WT-F/F/S: *n*=18, µMT-F/S: *n*=22, µMT-F/F/S: *n*=26). (**B)** Number of MRSA colonies from infected right upper mouse lung homogenates (WT-F/S *n*=12, WT-F/F/S: *n*=16, µMT-F/S: *n*=22, µMT-F/F/S: *n*=21). **(C)** Presence of viral protein (PR8, Matrix Protein, M1) was assessed via qPCR (WT-F/S *n*=12, WT-F/F/S: *n*=9, µMT-F/S: *n*=12, µMT-F/F/S: *n*=12). **(D)** Blinded histology scores of parenchymal lung tissue sections (WT-F/S *n*=14, WT-F/F/S: *n*=18, µMT-F/S: *n*=14, µMT-F/F/S: *n*=18). Data represented as mean ± SEM and *P* values were determined by repeated 1-way ANOVA measures (**p* < 0.05, ***p* < 0.01, ****p* < 0.001, *****p* < 0.0001).

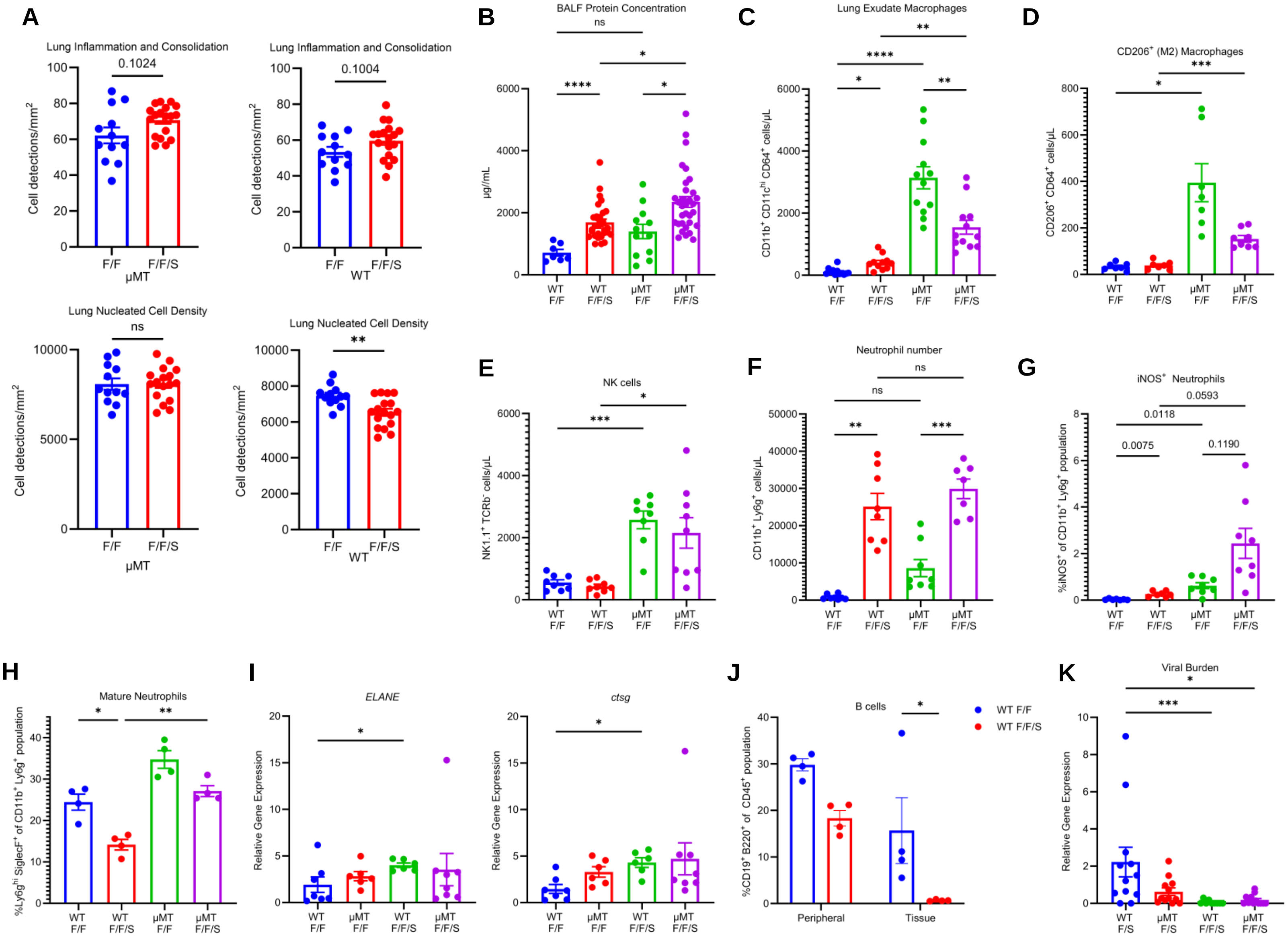

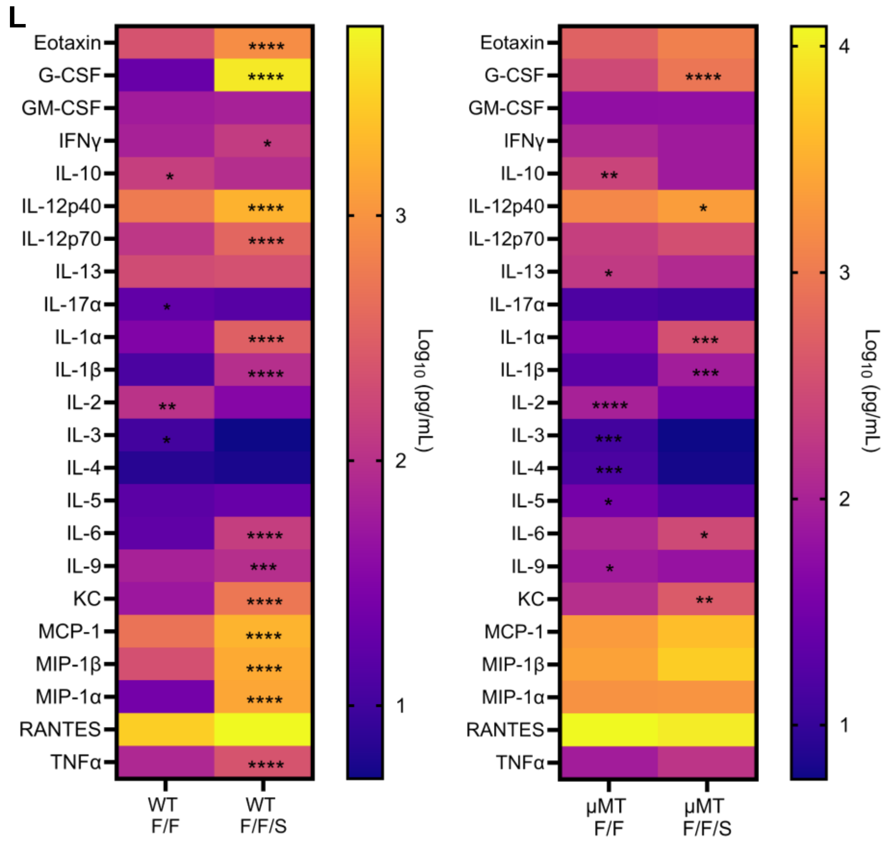

SUPPLEMENTAL FIGURE 2. MRSA challenge leads to more lung tissue injury, increases inflammatory cytokines and alters immune cell populations. **(A)** Qupath was used to quantify lung nucleated cell density (cells/mm^2) (WT-F/F: *n*=12, WT-F/F/S: *n*=17, μMT-F/F: *n*=12, μMT-F/F/S: *n*=17) and frequency of lung inflammation and consolidation detections (WT-F/F: *n*=12, WT-F/F/S: *n*=18, μMT-F/F: *n*=12, μMT-F/F/S: *n*=18) between F/F versus F/F/S treatment groups. **(B)** Protein was quantified from BALF and was collected at day of harvest (WT-F/F: *n*=7, WT-F/F/S: *n*=30, μMT-F/F: *n*=12, μMT-F/F/S: *n*=32). **(C)** Absolute number of CD64$^+$CD11b$^+$CD11c^{hi} cells (WT-F/F: *n*=12, WT-F/F/S: *n*=11, μMT-F/F: *n*=12, μMT-F/F/S: *n*=12). **(D)** Absolute number of CD64$^+$CD206$^+$ cells (WT-F/F: *n*=8, WT-F/F/S: *n*=7, μMT-F/F: *n*=7, μMT-F/F/S: *n*=8). **(E)** Absolute number of NK1.1$^+$TCRβ$^-$ cells (WT-F/F: *n*=8, WT-F/F/S: *n*=8, μMT-F/F: *n*=8, μMT-F/F/S: *n*=9). **(F)** Absolute number of CD11b$^+$Ly6g$^+$ cells (WT-F/F: *n*=8, WT-F/F/S: *n*=8, μMT-F/F: *n*=8, μMT-F/F/S: *n*=7). **(G)** Percentage of iNOS$^+$CD11b$^+$Ly6g$^+$ cells (WT-F/F: *n*=7, WT-F/F/S: *n*=7, μMT-F/F: *n*=8, μMT-F/F/S: *n*=8). **(H)** Percentage of Ly6g^{hi}SiglecF$^+$CD11b$^+$ cells (WT-F/F: *n*=4, WT-F/F/S: *n*=4, μMT-F/F: *n*=4, μMT-F/F/S: *n*=4). **(I)** Relative expression of genes related to neutrophil function (*ELANE, ctsg*) calculated via qPCR (WT-F/F: *n*=6, WT-F/F/S: *n*=7, μMT-F/F: *n*=8, μMT-F/F/S: *n*=6). **(J)** Intravascular staining shows tissue vs. peripheral percentages of CD19$^+$B220$^+$ B cells (WT-F/F: *n*=4, WT-F/F/S: *n*=4). **(K)** Presence of viral protein (PR8) was assessed via qPCR (WT-F/F: n=8, WT-F/F/S: n=7, μMT-F/F: n=7, μMT-F/F/S: n=8). **(L)** Heatmap displaying the protein expression of 23 cytokines from lung homogenate comparing F/F and F/F/S infections for WT and μMT mice cells (WT-F/F: *n*=12, WT-F/F/S: *n*=26, μMT-F/F: *n*=12, μMT-F/F/S: *n*=22). Data represented as mean ± SEM. For panels **A & L**, *P* values were determined by repeated 2-tailed Mann-Whitney U-test. For panels **B-K**, *P* values were determined by repeated 1-way ANOVA measures (*$p < 0.05$, **$p < 0.01$, ***$p < 0.001$, ****$p < 0.0001$).

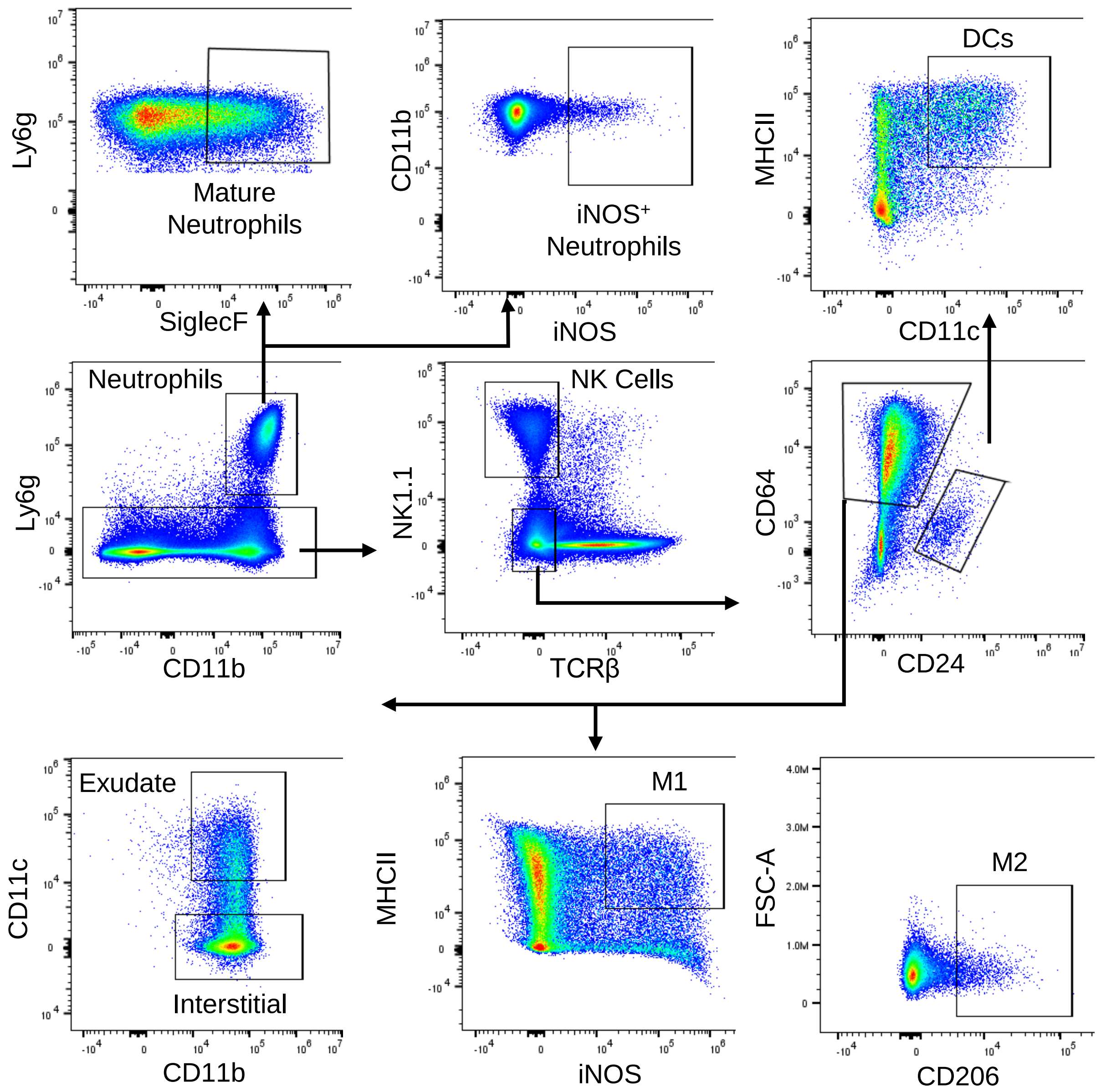

SUPPLEMENTAL FIGURE 3. Gating strategy for myeloid compartment analysis. Samples were initially gated on live, single, CD45+ CD19- cells and further gated to analyze phenotypic changes of innate cells.

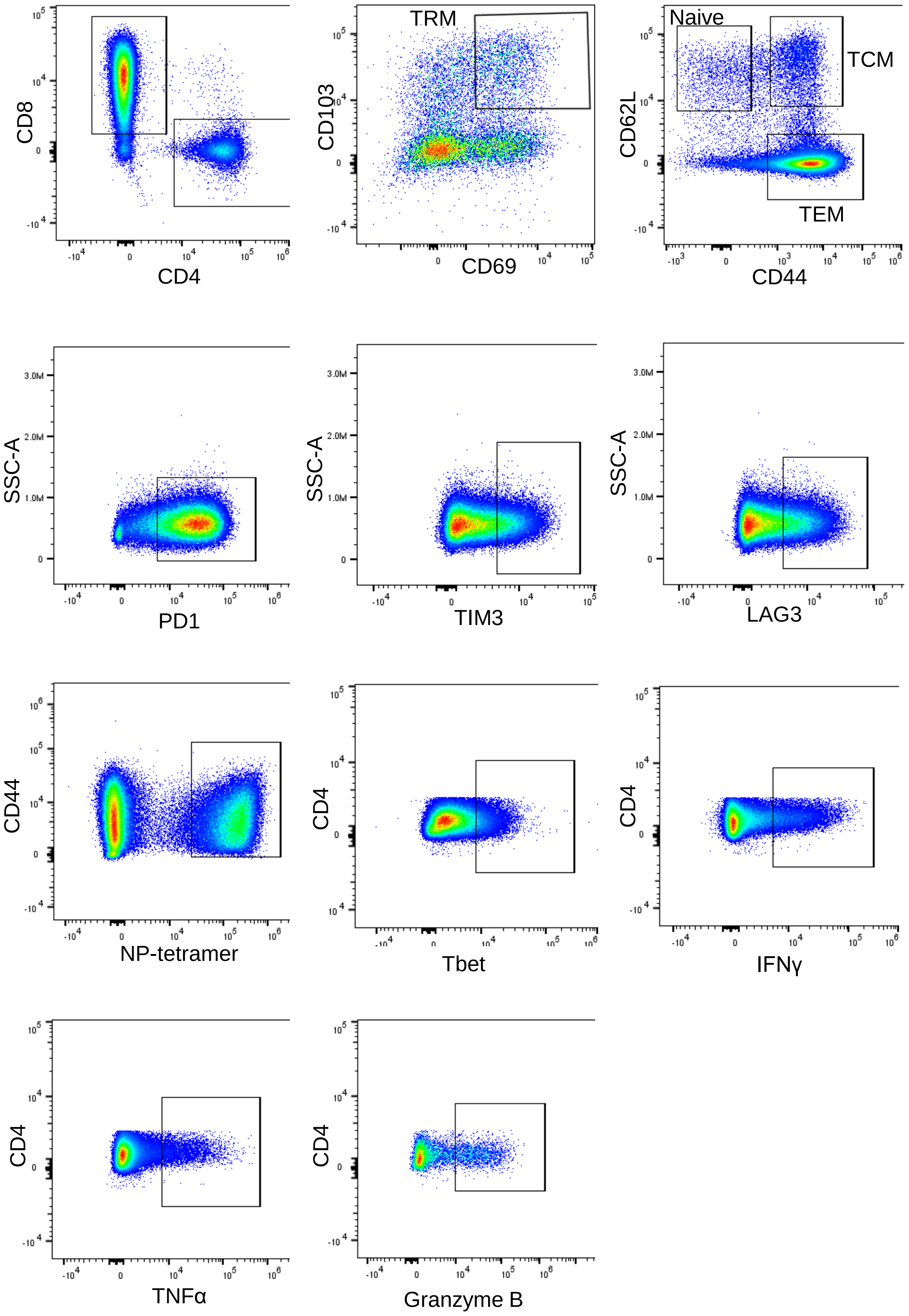

SUPPLEMENTAL FIGURE 4. Gating strategy for T cell compartment analysis. Samples were initially gated on live, single, $CD45^{+}$ $B220^{-}$ $CD11b^{-}$ $CD11c^{-}$ $CD90.2^{+}$ cells and further gated to analyze phenotypic and functional changes in $CD8^{+}$ T cells.

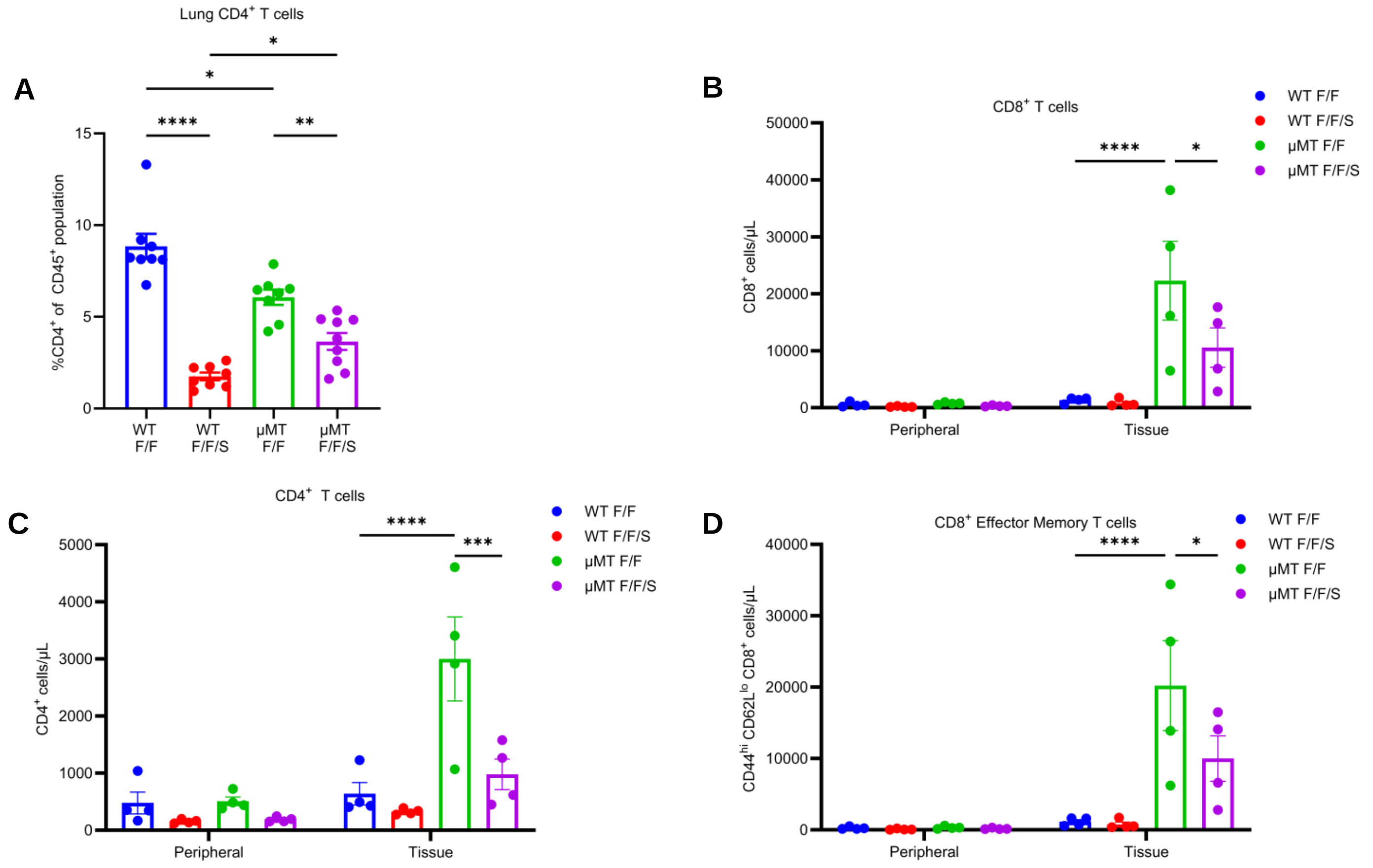

SUPPLEMENTAL FIGURE 5. T cells of B cell deficient mice are located primarily in the lung tissue. **(A)** Conventional flow cytometry shows the absolute number of $CD4^+$ T cells (WT-F/F: *n*=8, WT-F/F/S: *n*=7, µMT-F/F: *n*=8, µMT-F/F/S: *n*=8). Intravascular staining shows tissue vs. peripheral immune cell populations. **(B-D)** Absolute number of $CD8^+$ T cells, absolute number of $CD4^+$ T cells, and absolute number of $CD44^{hi}CD62^{lo}CD8^+$ T cells (WT-F/F: *n*=4, WT-F/F/S: *n*=4, µMT-F/F: *n*=4, µMT-F/F/S: *n*=4). Data represented as mean ± SEM and *P* values were determined by 1-way (Panel A) or 2-way (Panels B-D) ANOVA measures (*p < 0.05, ***p <0.001, ****p < 0.0001).

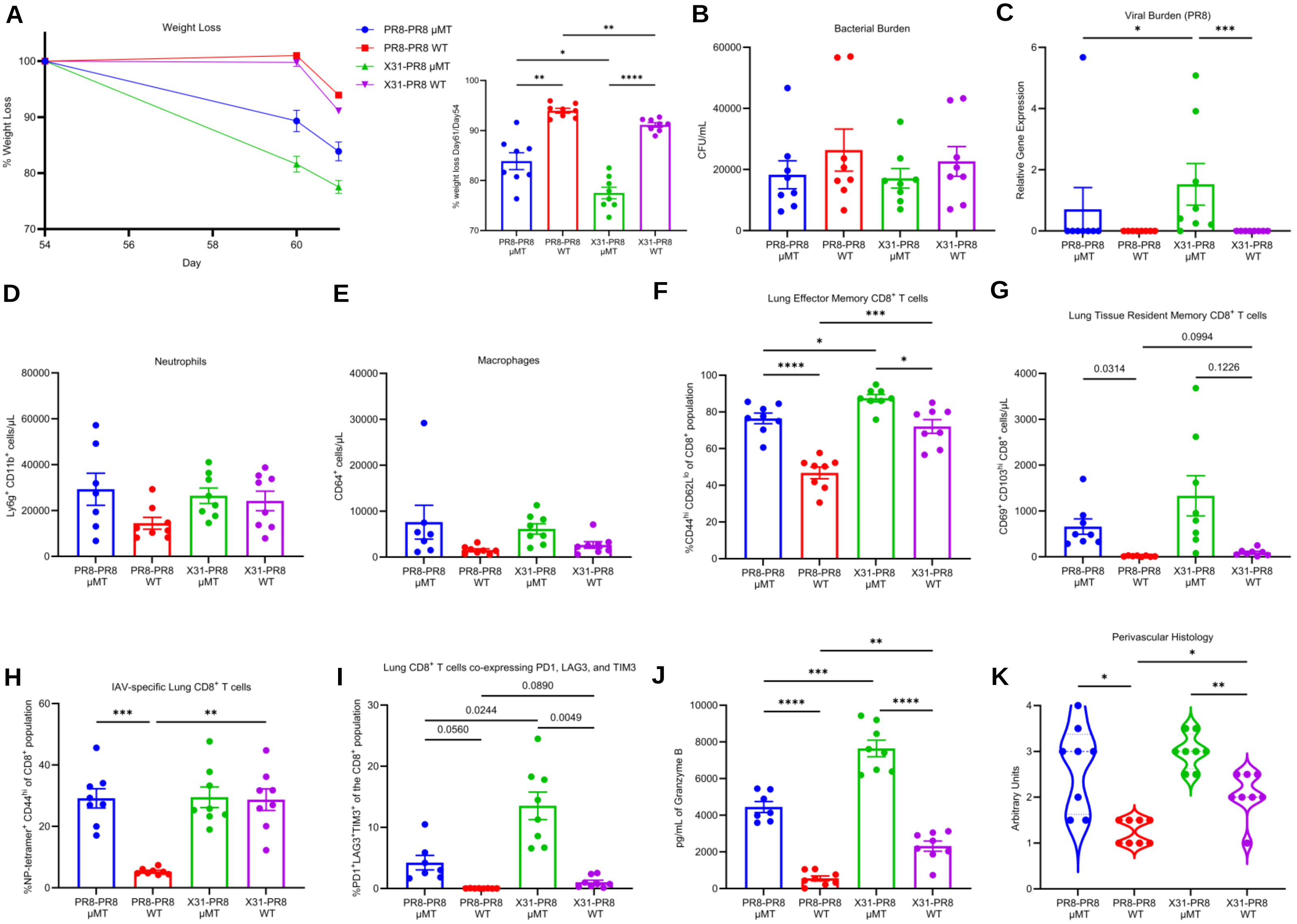

SUPPLEMENTAL FIGURE 6. Heterotypic influenza challenge increased weight loss, viral burden, and CD8⁺ T cell activation compared to homotypic influenza infection. **(A)** Percent of weight loss was calculated starting from secondary infection with PR8 for each mouse strain (µMT-PR8-PR8: *n*=8, WT-PR8-PR8: *n*=8, µMT-X31-PR8: *n*=8, WT-X31-PR8: *n*=8). **(B)** Number of MRSA colonies plates from infected right upper mouse lung homogenates (µMT-PR8-PR8: *n*=8, WT-PR8-PR8: *n*=8, µMT-X31-PR8: *n*=8, WT-X31-PR8: *n*=8). **(C)** Presence of viral protein (PR8) was assessed via qPCR (µMT-PR8-PR8: *n*=8, WT-PR8-PR8: *n*=8, µMT-X31-PR8: *n*=8, WT-X31-PR8: *n*=8). **(D)** Absolute number of $CD11b^{+}Ly6g^{+}$ cells (µMT-PR8-PR8: *n*=7, WT-PR8-PR8: *n*=8, µMT-X31-PR8: *n*=8, WT-X31-PR8: *n*=8). **(E)** Absolute number of $CD64^{+}CD45^{+}TCR\beta^{-}NK1.1^{-}Ly6g^{-}$ cells (µMT PR8-PR8: *n*=7, WT-PR8-PR8: *n*=8, µMT-X31-PR8: *n*=8, WT-X31-PR8: *n*=8). **(F)** Percentage of $CD44^{hi}CD62^{lo}CD8^{+}$ cells (µMT-PR8-PR8: *n*=8, WT-PR8-PR8: *n*=8, µMT-X31-PR8: *n*=8, WT-X31-PR8: *n*=8). **(G)** Absolute number of $CD69^{+}CD103^{hi}CD8^{+}$ cells (µMT-PR8-PR8: *n*=8, WT-PR8-PR8: *n*=8, µMT-X31-PR8: *n*=8, WT-X31-PR8: *n*=8). **(H)** Percentage of NP-tetramer^{+}CD44^{hi}CD8^{+} cells (µMT-PR8-PR8: *n*=8, WT-PR8-PR8: *n*=8, µMT-X31-PR8: *n*=8, WT-X31-PR8: *n*=8). **(I)** Percentage of PD-1^{+}LAG-3^{+}TIM-3^{+}CD8^{+} cells (µMT-PR8-PR8: *n*=7, WT-PR8-PR8: *n*=8, µMT-X31-PR8: *n*=8, WT-X31-PR8: *n*=8). **(J)** Protein expression of Granzyme B from BALF (µMT-PR8-PR8: *n*=7, WT-PR8-PR8: *n*=8, µMT-X31-PR8: *n*=8, WT-X31-PR8: *n*=8). **(K)** Blinded histology scores of perivascular lung tissue sections (µMT-PR8-PR8: *n*=8, WT-PR8-PR8: *n*=8, µMT-X31-PR8: *n*=8, WT-X31-PR8: *n*=8). Data represented as mean ± SEM and *P* values were determined by repeated 1-way ANOVA measures (*$p < 0.05$, **$p < 0.01$, ***$p < 0.001$, ****$p < 0.0001$).

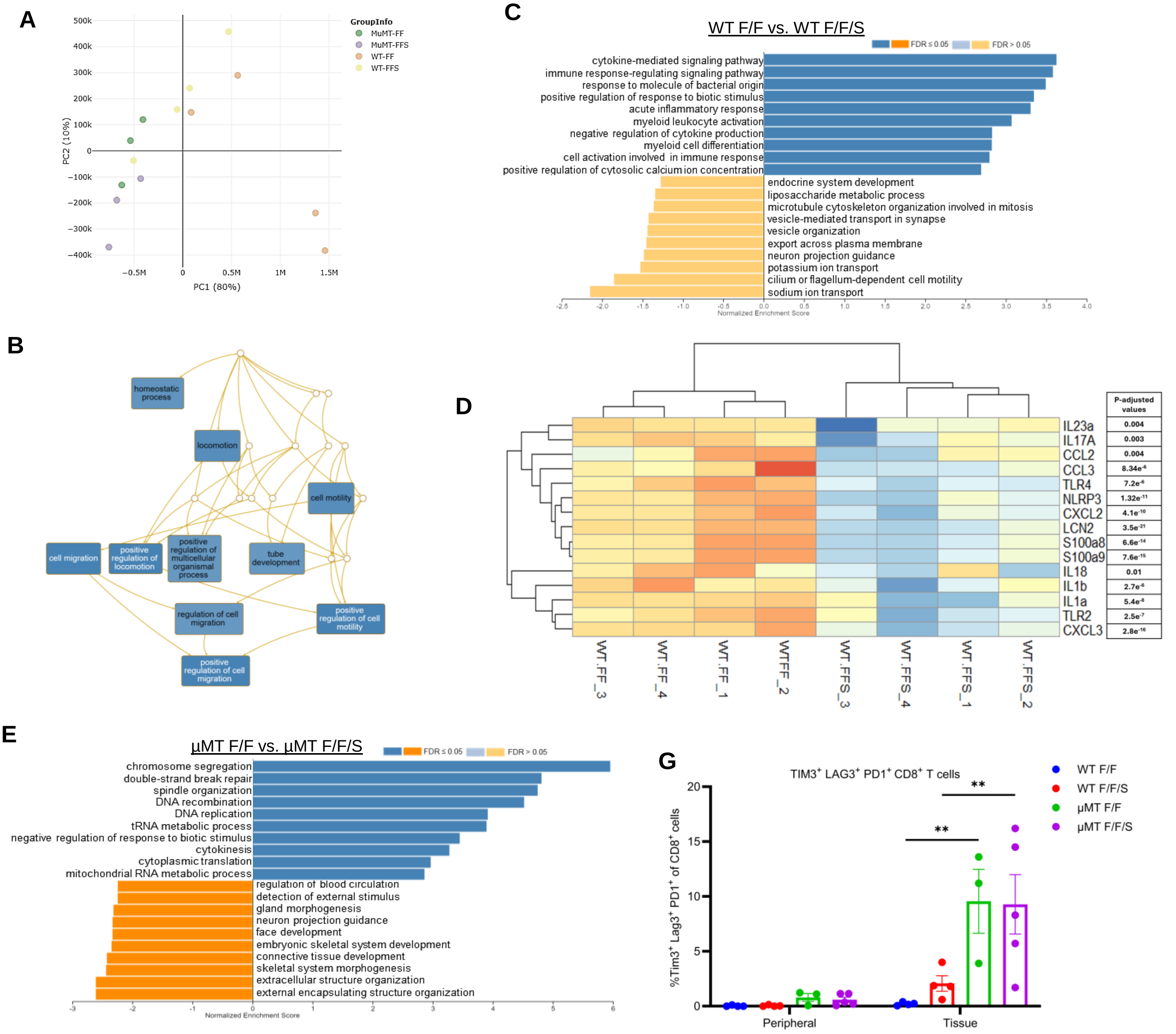

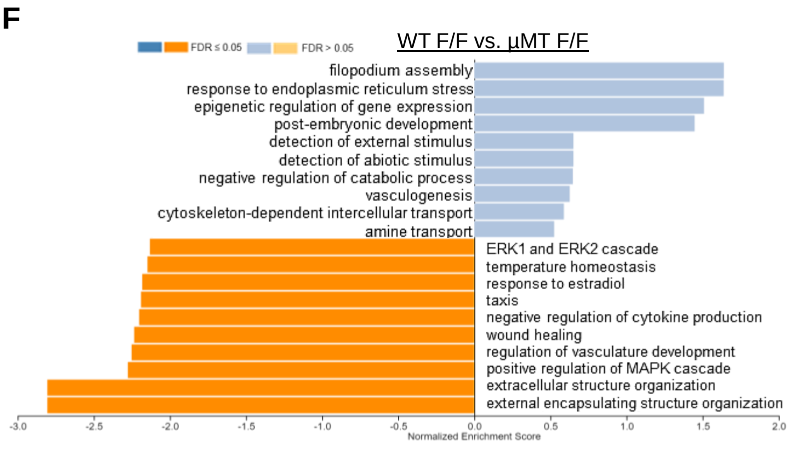

SUPPLEMENTAL FIGURE 7. Bulk-RNA-seq analyses of sorted lung CD8$^+$ T cells from WT and μMT mice with either F/F or F/F/S infections (WT-F/F: *n*=4, WT-F/F/S: *n*=4, μMT-F/F: *n*=3, μMT-F/F/S: *n*=3). **(A)** Principal-component analysis plot showing how the groups (WT F/F, WT F/F/S, μMT F/F, μMT F/F/S) cluster. **(B)** GO network analysis of differentially expressed genes (adjusted *p*-value < 0.05) shared between WT F/F vs. WT F/F/S and μMT F/F vs. μMT F/F/S analyses. **(C)** Top and bottom 10 GO biological pathways in WT F/F vs. WT F/F/S on genes with false discovery rate *p*-adjusted values < 0.05. **(D)** Clustering heatmap of log$_2$ transformed TPM values of WT F/F and WT F/F/S mice for several genes related to anti-bacterial immunity with adjusted *p*-values (right). **(E)** Top and bottom 10 GO biological pathways in μMT F/F vs μMT F/F/S on genes with false discovery rate *p*-adjusted values < 0.05. **(F)** Top and bottom 10 GO biological pathways in WT F/F vs. μMT F/F on genes with false discovery rate *p*-adjusted values < 0.05. **(G)** Intravascular staining shows tissue vs. peripheral percentages of PD-1$^+$CD8$^+$ T cells (WT-F/F: *n*=4, WT-F/F/S: *n*=4, μMT-F/F: *n*=4, μMT-F/F/S: *n*=4). Data represented as mean ± SEM and *P* values were determined by 2-way ANOVA measures (***p* < 0.01).

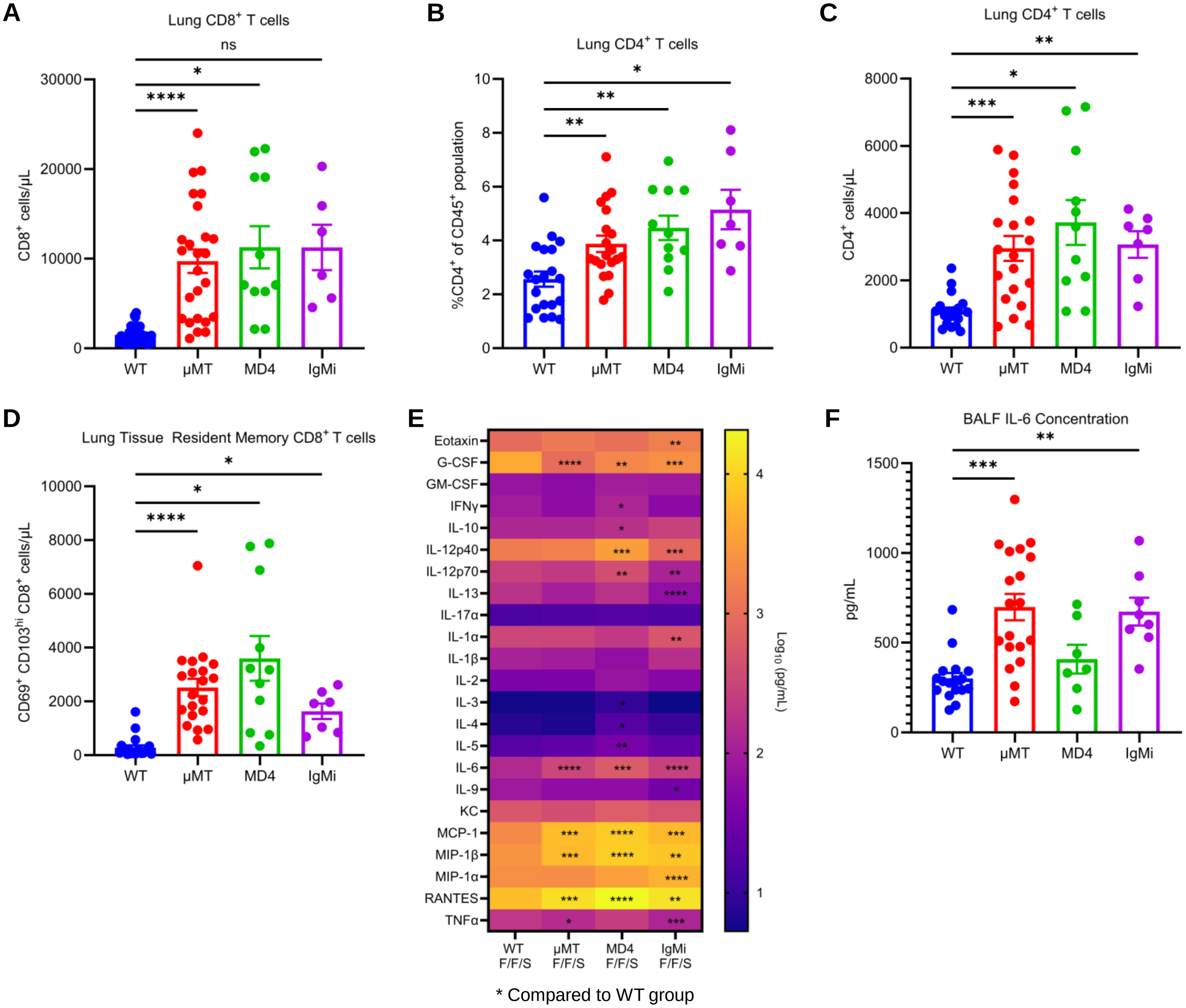

SUPPLEMENTAL FIGURE 8. Loss of influenza virus-specific antibody alters cytotoxic memory $CD8^+$ T cell responses. **(A)** Flow cytometry analysis on WT, µMT, MD4, and IgMi lungs. Absolute number of $CD8^+$ T cells (WT: *n*=30, MD4: *n*=11, µMT: *n*=25, IgMi: *n*=6). **(B-C)** Percentage and absolute number of $CD4^+$ T cells (WT: *n*=20, MD4: *n*=11, µMT: *n*=20, IgMi: *n*=7). **(D)** Absolute number of $CD69^+CD103^{hi}CD8^+$ T cells (WT: *n*=20, MD4: *n*=11, µMT: *n*=20, IgMi: *n*=7). **(E)** Heatmap displaying the protein expression of 23 cytokines from lung homogenate. Statistical significance compares B cell deficient groups (µMT, MD4, IgMi) to WT (WT: *n*=26, MD4: *n*=14, µMT: *n*=22, IgMi: *n*=8). **(F)** Protein expression of IL-6 from BALF (WT: *n*=17, MD4: *n*=7, µMT: *n*=19, IgMi: *n*=8). Data represented as mean ± SEM and *P* values were determined by repeated 1-way ANOVA measures (*p < 0.05, **p < 0.01, ***p <0.001, ****p < 0.0001).

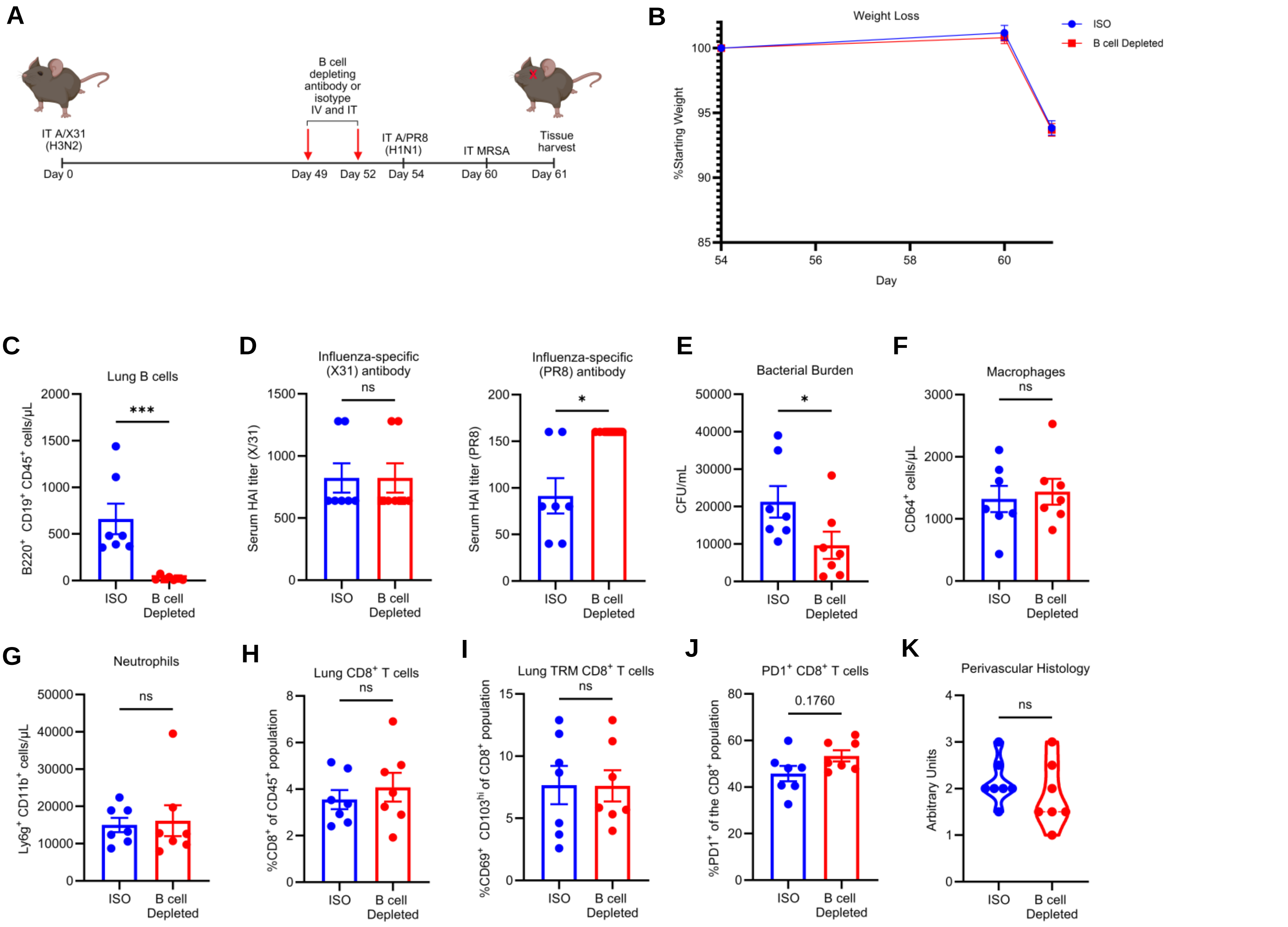

SUPPLEMENTAL FIGURE 9. Depleting B cells prior to secondary influenza infection did not impact $CD8^+$ T cell phenotype or control of super-infection **(A)** WT mice were administered CD20-depleting antibody (SA271G2, Biolegend) or IgG2b isotype antibody intravenously and intratracheally 5 and 2 days prior to PR8 challenge to deplete B cells during secondary influenza virus infection. **(B)** Percent of weight loss was calculated starting from secondary infection with PR8 for each treatment group. **(C)** Flow cytometry was performed on lung tissue. Absolute number of $B220^+CD19^+CD45^+$ cells. **(D)** X-31 or PR8 influenza virus-specific mouse serum titers measured by HAI assay at day of tissue harvest. **(E)** Number of MRSA colonies plated from infected right upper mouse lung homogenates**.** **(F)** Absolute number of lung $CD64^+CD24^-CD45^+CD19^-TCR\beta^-NK1.1^-$ cells. **(G)** Absolute number of lung $Ly6g^+CD11b^+CD45^+$ cells. **(H)** Frequency of lung $CD8^+$ cells of $CD45^+$ population **(I)** Frequency of $CD69^+CD103^{hi}$ cells of $CD8^+$ population. **(J)** Frequency of lung $PD1^+$ cells of $CD8^+$ population. **(K)** Blinded histology scores of perivascular lung tissue sections. For all figures, (ISO: n=7, B cell-depleted n=7). Data represented as mean ± SEM and P values were determined by repeated Mann-Whitney U-test ($^*p < 0.05$, $^{**}p < 0.01$).

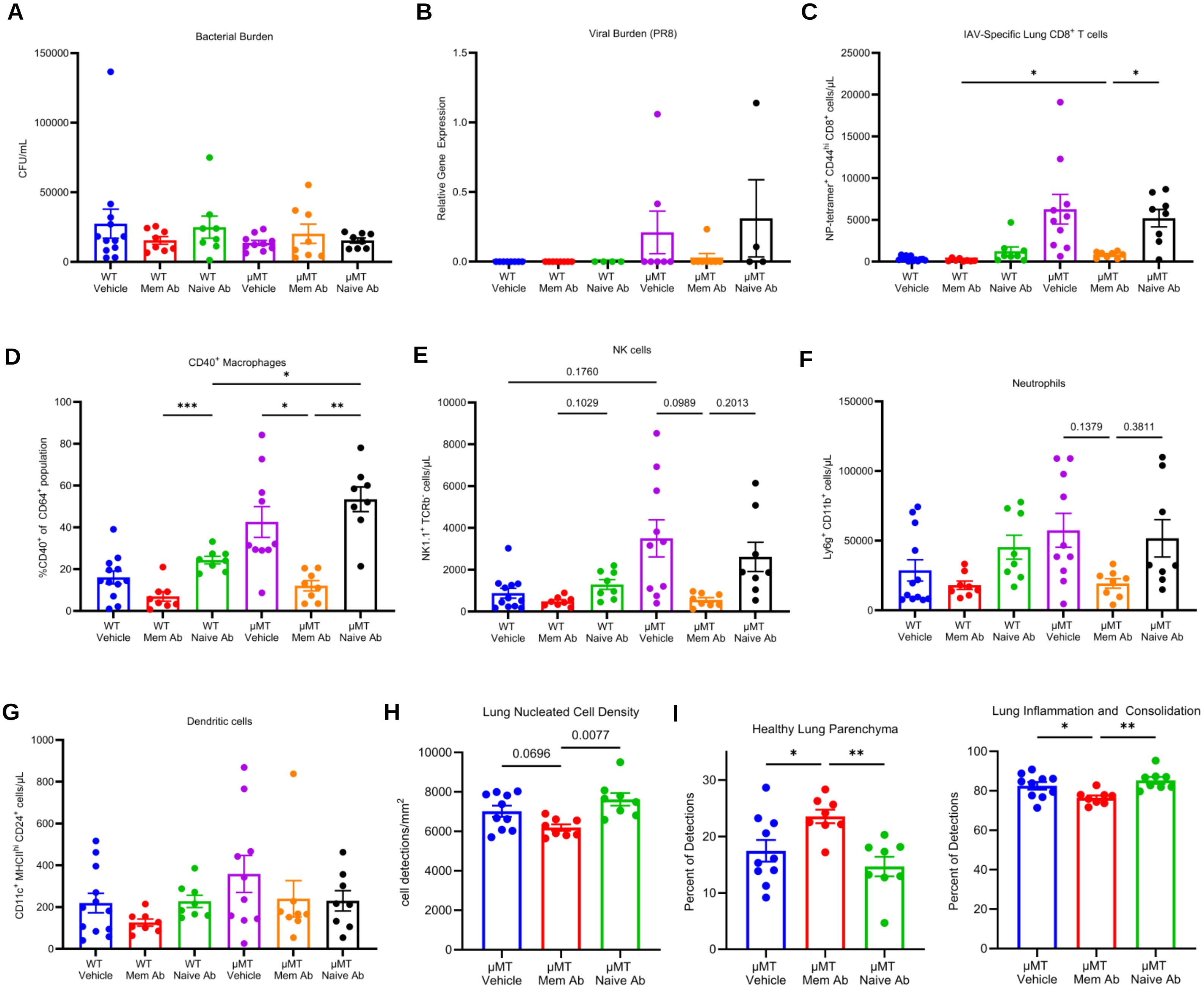

SUPPLEMENTAL FIGURE 10. Passive immunization with memory serum impacts recruitment and activation of T cells and macrophages **(A)** Number of MRSA colonies plated from infected right upper mouse lung homogenates (WT-vehicle: *n*=12, WT-memory-Ab: *n*=8, WT-naïve-Ab: *n*=8, µMT-vehicle: *n*=10, µMT-memory-Ab: *n*=8, µMT-naïve-Ab: *n*=8). **(B)** Presence of viral protein (PR8) was assessed via qPCR (WT-vehicle: *n*=8, WT-memory-Ab: *n*=8, WT-naïve-Ab: *n*=4, µMT-vehicle: *n*=7, µMT-memory-Ab: *n*=8, µMT-naïve-Ab: *n*=4). **(C)** Flow cytometry was completed on lungs and all populations were initially gated on live, single cells. Absolute number of lung NP-tetramer^{+}CD44^{hi}CD8^{+} cells (WT-vehicle: *n*=12, WT-memory-Ab: *n*=8, WT-naïve-Ab: *n*=8, µMT-vehicle: *n*=10, µMT-memory-Ab: *n*=8, µMT-naïve-Ab: *n*=8). **(D)** Percentage of lung CD40^{+} cells of CD64^{+} population (WT-vehicle: *n*=12, WT-memory-Ab: *n*=8, WT-naïve-Ab: *n*=8, µMT-vehicle: *n*=10, µMT-memory-Ab: *n*=8, µMT-naïve-Ab: *n*=8). **(E)** Absolute number of lung NK1.1^{+}TCRβ^{-}Ly6g^{-}CD45^{+} cells (WT-vehicle: *n*=12, WT-memory-Ab: *n*=8, WT-naïve-Ab: *n*=8, µMT-vehicle: *n*=10, µMT-memory-Ab: *n*=8, µMT-naïve-Ab: *n*=8). **(F)** Absolute number of lung Ly6g^{+}CD11b^{+}CD45^{+} cells (WT-vehicle: *n*=12, WT-memory-Ab: *n*=8, WT-naïve-Ab: *n*=8, µMT-vehicle: *n*=10, µMT-memory-Ab: *n*=8, µMT-naïve-Ab: *n*=8). **(G)** Absolute number of lung CD11c^{+}MHCII^{hi}CD24^{+}CD64^{+}CD45^{+}CD19^{-}TCRβ^{-} cells (WT-vehicle: *n*=12, WT-memory-Ab: *n*=8, WT-naïve-Ab: *n*=8, µMT-vehicle: *n*=10, µMT-memory-Ab: *n*=8, µMT-naïve-Ab: *n*=8). **(H)** Qupath was used to quantify lung nucleated cell density (cells/mm^{2}) for treatment groups (µMT-vehicle: *n*=10, µMT-memory-Ab: *n*=8, µMT-naïve-Ab: *n*=8). **(I)** Qupath was used to calculate the frequency of healthy lung parenchyma (left) and frequency of lung inflammation and consolidation detections (right) (µMT-vehicle: *n*=10, µMT-memory-Ab: *n*=8, µMT-naïve-Ab: *n*=8). Data represented as mean ± SEM and *P* values were determined by repeated 1-way ANOVA measures (**p* < 0.05, ***p* < 0.01, ****p* <0.001).

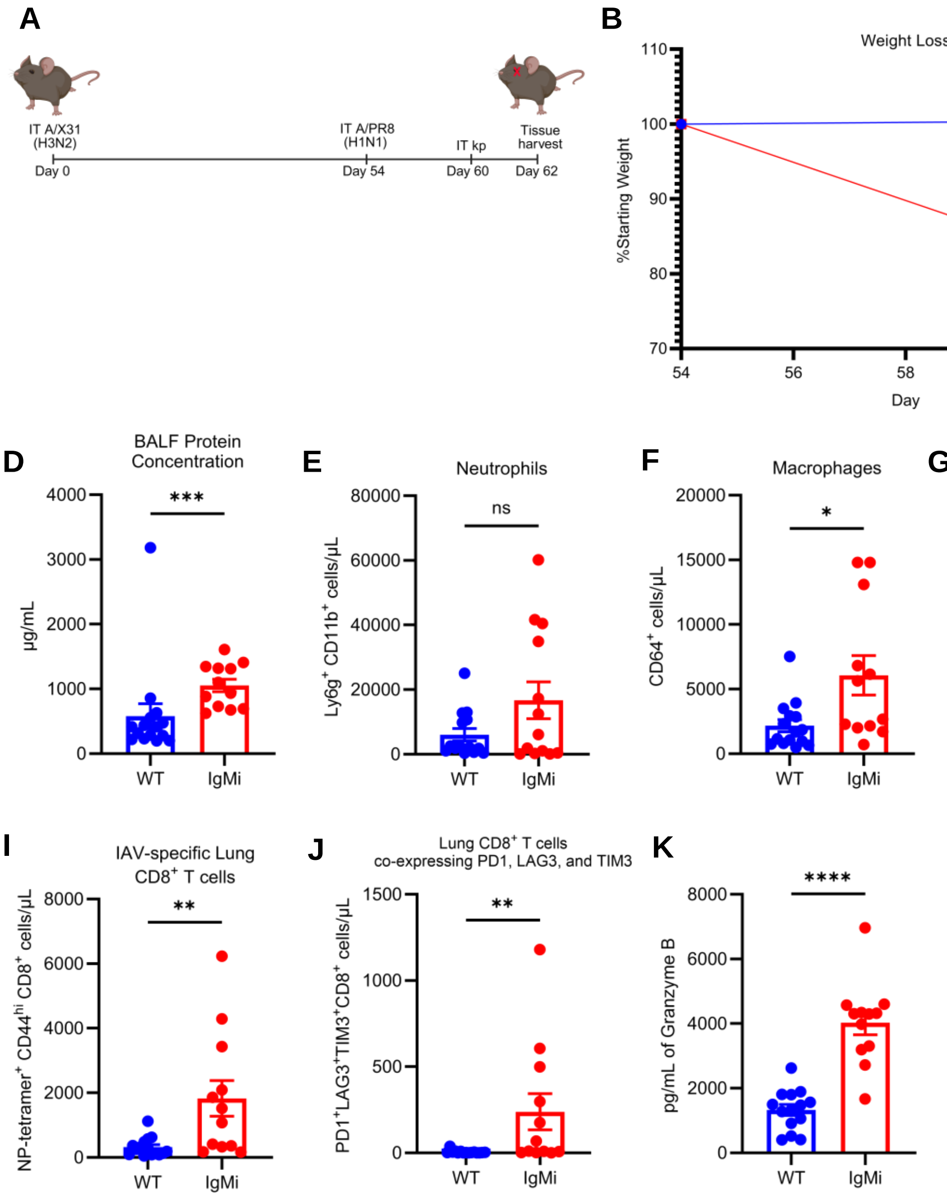

SUPPLEMENTAL FIGURE 11. Heterotypic influenza super-infection with *klebsiella pnuemoniae* resulted in increased memory CD8$^+$ T cells in the lung **(A)** Infection scheme for F/F/KP challenge: mice were challenged with X/31 at day 0, challenged with PR8 at day 54, and challenged with *klebsiella pnuemoniae* (5000 CFU) at day 60. Tissue harvest was 48 hours following KP challenge. **(B)** Percent of weight loss was calculated starting from secondary infection with PR8 for each treatment group. **(C)** Number of KP colonies plated from infected right upper mouse lung homogenates. **(D)** Protein was quantified from BALF and was collected at day of harvest. **(E)** Flow cytometry was performed with lung tissue and all populations were initially gated on live, single cells. Absolute number of Ly6g$^+$CD11b$^+$CD45$^+$ cells. **(F)** Absolute number of CD64$^+$CD24$^-$CD45$^+$ cells in the lung. **(G)** Absolute number of lung CD8$^+$CD90.2$^+$CD45$^+$ cells. **(H)** Absolute number of lung CD69$^+$CD103^{hi}CD8$^+$ cells. **(I)** Absolute number of lung NP-tetramer$^+$CD44^{hi}CD8$^+$ cells. **(J)** Absolute number of lung PD1$^+$LAG3$^+$TIM3$^+$CD8$^+$ cells **(K)** Protein expression of Granzyme B from BALF. For all figures, (WT n=13, IgMi n=12). Data represented as mean ± SEM and P values were determined by repeated Mann-Whitney U-test (*p < 0.05, **p < 0.01, ***p <0.001, ****p < 0.0001).

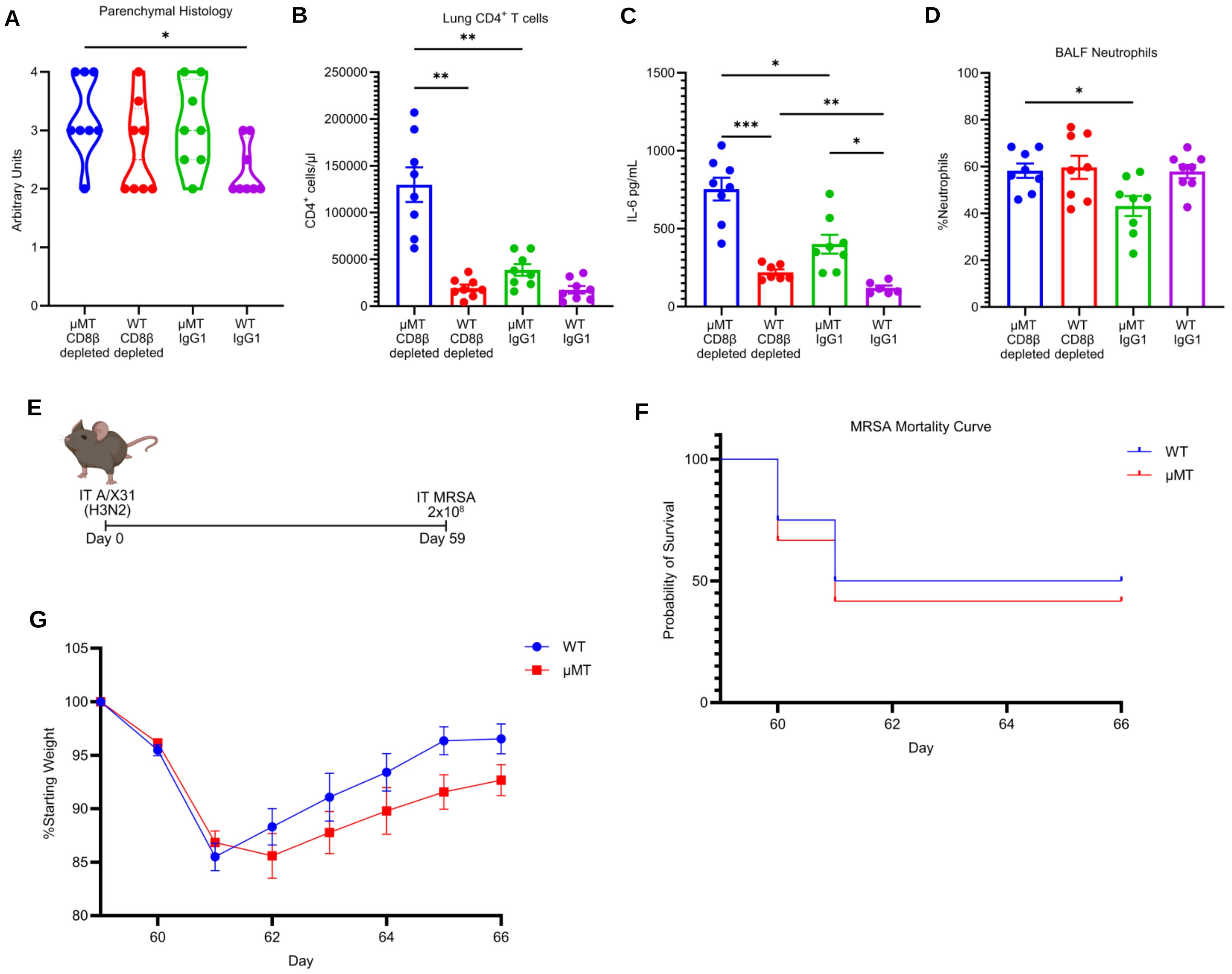

SUPPLEMENTAL FIGURE 12. CD8β T cell depletion increased other immune cell subsets in the lung. **(A)** Blinded histology scores of parenchymal lung tissue sections (μMT-CD8β-depleted: *n*=8, WT-CD8β-depleted: *n*=8, μMT-ISO: *n*=8, WT-ISO *n*=8). **(B)** Flow cytometry was performed on mouse lungs. Absolute cell counts of $CD4^+$ T cells (μMT-CD8β-depleted: *n*=8, WT-CD8β-depleted: *n*=8, μMT-ISO: *n*=8, WT-ISO *n*=8). **(C)** IL-6 protein concentration was measured from BALF (μMT-CD8β-depleted: *n*=8, WT-CD8β-depleted: *n*=7, μMT-ISO: *n*=8, WT-ISO *n*=6). **(D)** Frequency of neutrophils from BALF (μMT-CD8β-depleted: *n*=8, WT-CD8β-depleted: *n*=8, μMT-ISO: *n*=8, WT-ISO *n*=8). **(E)** Infection scheme for lethal MRSA challenge: mice were challenged with X/31 at day 0 and administered a lethal dose of MRSA ($2x10^8$) 59 days later. **(F-G)** WT and μMT mice were checked for mortality and weighed daily to calculate percent weight loss. Data represented as mean ± SEM and *P* values were determined by repeated 1-way ANOVA measures (*$p < 0.05$, **$p < 0.01$, ***$p < 0.001$).

SUPPLEMENTAL METHODS

Sex as a biological variable

This study examined predominately male animals to reduce experimental variability. We have done some experiments (as indicated) with female mice and similar findings are reported for both sexes.

Mice

Six- to eight-week-old male wild-type (WT) C57BL/6 and MD4 (C57BL/6-Tg(IghelMD4)4Ccg/J) mice and were purchased from Jackson Laboratories (Bar Harbor, ME). Six- to eight-week-old male and female µMT (B6.129S2-Ighmtm1Cgn/J) were purchased from Jackson Laboratories (Bar Harbor, ME) or bred within the facilities at UPMC Children's Hospital of Pittsburgh. IgMi mice were a gift from the laboratory of Dr. Timothy Hand at UPMC Children's Hospital of Pittsburgh. Mice were maintained under pathogen-free conditions within the animal facilities of UPMC Children's Hospital of Pittsburgh. All studies were performed on sex- and age- matched mice. Animal studies were conducted with approval from the University of Pittsburgh Institutional Animal Care and Use Committee (protocol #23073501).

Mouse model and sampling collection

On day 0, 6- to 8- week male or female mice were infected with 0.5-1 x 10^5 PFU of mouse-adapted influenza virus A/HKx31 H3N2 (X31) or PBS vehicle. After 54 days, the mice were rechallenged with 10^3 PFUs of a heterotypic strain of mouse-adapted influenza virus A/PR/8/34 H1N1 (PR8) or PBS vehicle. Six days after influenza virus rechallenge (day 60), the mice were challenged with 5 x 10^7 CFUs of USA300 MRSA

(stationary phase) suspended in PBS or vehicle and harvested a day later. For homotypic influenza virus challenge models, mice were infected with PR8 at both viral challenge time points. For *Klebsiella pnuemoniae* experiments, mice were challenged with $5x10^3$ CFU of *K.pneumoniae* (ATCC 43816) at day 60 and tissues were harvested 48 hours later (day 62). All infections were given via oropharyngeal instillation. Mice were euthanized via pentobarbital injection followed by cervical dislocation and exsanguination by severing the renal artery.

Bronchoalveolar lavage fluid collection and differential cell staining

At harvest, mice were cannulated and lavaged with 1mL of PBS for bronchoalveolar lavage (BAL) fluid (BALF) collection. BALF was centrifuged (2,000g, 5 min) to pellet cells and collect supernatant for downstream analysis. Supernatant was stored at -80°C. Pelleted cells were treated with ACK lysis buffer (Life Technologies Fisher Scientific, Hampton, NH) to remove RBCs. The pellet was then resuspended in 500µl- 1mL of PBS and total cell count was determined by hemacytometer or by a TC20 automated cell counter (Bio-Rad, Hercules, CA). A total of 200µL of resuspended cells was then concentrated on a microscope slide using a cytospin centrifuge (Thermo Fisher Scientific, Waltham, MA). The slides were then stained with Hema 3 Solution II staining solutions (Fisher Scientific, Hampton, NH) to determine the counts of neutrophils, monocytes/macrophages, eosinophils, and lymphocytes.

Bacterial plating

The upper right lung lobes of mice were collected and mechanically homogenized in 1 mL of PBS. Neat and 10-fold dilutions of the lung homogenate were dot plated on culture plates. Plates were then incubated at 37°C overnight and then CFUs were quantified by bacterial colony counting.

Flow cytometry

Mouse lung lobes were aseptically dissected and minced with sterile scissors and incubated in 10 mL of collagenase media (DMEM; Life Technologies Fisher Scientific, Hampton, NH, Collagenase IV; Worthington Biochemical, Lakewood, NJ, DNase I; Sigma-Aldrich, St. Louis, MO) for 1 hour shaking at 37°C. After the incubation, the lungs were smashed through a 70-μm filter and rinsed with DMEM media with 10% fetal bovine serum (FBS) to obtain a single cell suspension. The single cell suspension was centrifuged (800g, 8 min) and the cell pellet was treated with ACK lysis buffer (Life Technologies Fisher Scientific, Hampton, NH) to remove RBCs. Cells were then resuspended in PBS and cell counts were obtained by hemacytometer or by a TC20 automated cell counter (Bio-Rad, Hercules, CA). Single cell suspensions were stained with antibodies (Table 1) as follows for spectral flow cytometry. The viability dyes, Zombie NIR (Biolegend, San Diego, CA) or LiveDead Blue (ThermoFisher, Invitrogen, Carlsbad, CA) were used to exclude dead cells from live cells in the T cell or myeloid panels. Mouse CD16/CD32 Fc Block (BD Biosciences, Franklin Lakes, NJ) was used during viability staining. The master-mix for flow cytometry staining contained Super Bright Complete Staining Buffer (ThermoFisher Scientific, Waltham, MA) as well as True-Stain Monocyte Blocker (Biolegend, San Diego, CA) for the myeloid panel. For

intracellular staining, cells were fixed and permeabilized with the eBioscience Foxp3/Transcription Factor Staining Buffer Set (ThermoFisher Scientific, Waltham, MA) as directed by the manufacturer. Influenza virus-specific CD8 T cells were isolated via class I MHC tetramer staining with PE-labeled Tetramer [H2-Db Influneza A NP 366-374 ASNENMETM], which was obtained from the National Institutes of Health Tetramer Core Facility (Emory University, Atlanta, GA). For tetramer staining, single cell lung suspensions were stained in complete RPMI (Life Technologies Fisher Scientific, Hampton, NH) media for 30 minutes at 37°C in the dark prior to viability staining. For detection of apoptotic cells, single cell lung suspensions were stained with the BD Pharmigen PE Annexin V Apoptosis Detection Kit I (BD Biosciences, Franklin Lakes, NJ) according to manufacturer instructions. All samples were run on the Cytek Aurora (Cytek Biosciences, Freemont, CA). Flow cytometric analysis was performed with FlowJo (Ashland, OR) and CytoBank (Beckamn Coulter, Brea, CA) software. FlowJo software was used for manual gating and for concatenation of sample files. Cytobank software was used for tSNE and FlowSOM analysis of flow cytometry data. Absolute cell counts were determined using the lung single cell suspension counts and the Aurora Cytek flow rate.

Histology and Qupath analysis

Left lobes of mice were inflated with and preserved for at least 48 hours with 10% neutral-buffered formalin solution. After formalin-fixation, lobes were transferred to 70% ethanol. Mouse lung lobes were processed by the Histology Core of UPMC Children’s Hospital of Pittsburgh, where the samples were paraffin embedded and sectioned for histopathological analysis. On sectioning, H&E staining was performed. Histological

scoring was performed on H&E-stained slides using a scale from 1 to 4, with 1 being no damage and 4 being severely damaged. Cellular infiltration and tissue damage was assessed for the peribronchial, perivascular, and parenchymal regions. Scoring was performed on blinded slides by two separate investigators. H&E-stained lung sections were imaged at 40X magnification on a Leica Aperio CS2 digital slide scanner. The images were analyzed in QuPath (76), an open-source software used to analyze whole image slides. Qupath has built-in algorithms to detect cells and tissues, which can be classified using a machine learning based algorithm to identify objects using training datasets. For this study, control (uninfected) and influenza virus-infected lung sections were used to train Qupath 0.5.0 to identify healthy parenchyma, inflammation/immune cell infiltration, airways, and blood vessels. Lung sections were manually traced in the Qupath software, and the Qupath cell detection algorithm was applied to identify all lung nucleated cells. The trained machine learning algorithm was then applied to all lung sections included in this study to identify which lung nucleated cells were airways, blood vessels, healthy parenchyma, and inflammation/immune cell infiltration. These values were exported to Excel (Microsoft, Redmond, WA) to analyze the nucleated cell density (# of nucleated cells/mm^2) of the lung sections and to identify changes in the area of healthy versus inflamed parenchyma between study groups.

RNA extraction and quantitative PCR

The right middle mouse lung was isolated and snap frozen in liquid nitrogen, and stored at -80°C until later use. RNA was extracted as directed using the Qiagen RNeasy Mini Kit (Qiagen, Germantown, MD). cDNA was synthesized using the iScript cDNA

synthesis kit (Bio-Rad, Hercules, CA) as directed. Quantitative PCR was conducted using SsoAdvanced universal probe supermix (Bio-Rad, Hercules, CA) and target-specific FAM TaqMan real-time PCR assay primer probes (Thermo Fisher Scientific, Waltham, MA). Viral burden was determined by quantitative real-time RT-PCR on RNA extracted from lung tissue for the viral matrix protein (M1) as described previously (77,78). The following primers and probe were used for detection of M1 (forward primer: 5′-GGACTGCAGCGTAGACGCTT-3′, reverse primer: 5′-CATCCTGTTGTATATGAGGCCCAT-3′, probe: 5′-/56-FAM/CTCAGTTAT/ZEN/TCTGCTGGTGCACTTGCCA/3IABkFQ/−3’).

Hemagglutinin Inhibition Assay (HAI)

Hemagglutination inhibition assays were conducted on all serum samples. The serum samples were heat inactivated and tested for any non-specific agglutination with 0.5% turkey erythrocytes (Lampire Biological Laboratories, Pipersville, PA) incubated at room temperature for 30 minutes, vortexing every 10 minutes. Sera were then serially diluted twofold from 1:10 to 1:1280 and incubated with 4 hemagglutination units per 25 μL of the virus with 1% turkey erythrocytes in a V-bottom 96-well plate to quantify HAI titers. Hemagglutination inhibition titers were defined as the reciprocal of the last dilution of serum that completely inhibited hemagglutination (79).

Lincoplex and Protein assays

Protein levels in BALF were determined using the Pierce BCA protein assay kit (Thermo Fisher Scientific, Waltham, MA). Cytokine production was measured using

homogenate of upper right lung tissue (1 mL of PBS) using the Bio-Rad Magpix (Hercules, CA) multiplexing platform with the Bio-Plex Pro Mouse Cytokine 23-plex assay (Bio-Rad, Hercules, CA) as directed. BALF was used to determine granzyme B production using Mouse granzyme B DuoSet ELISA kit as directed (R&D Systems, Minneapolis, MN).

CD8β T cell depletion

CD8β T cell depletion was performed twice, five days before PR8 challenge (Day 49) and two days before PR8 challenge (Day 52). On the indicated days, μMT or WT C57BL/6 mice were injected intravenously via tail vein using a 29.5G syringe with 200 μg of *InVivo*MAb anti-mouse CD8β (Lyt 3.2, clone: 53-5.8, BioXcell, Lebanon, NH) or 200 μg of *InVivo*MAb rat IgG1 isotype control, anti-horseradish peroxidase (clone: HPRN, Biolegend, San Diego, CA). Then mice were oropharyngeally administered with 200 μg of *InVivo*MAb anti-mouse CD8β (Lyt 3.2, clone: 53-5.8, BioXcell, Lebanon, NH) or 200 μg of *InVivo*MAb rat IgG1 isotype control, anti-horseradish peroxidase (clone: HPRN, Biolegend, San Diego, CA). Depletion efficiency was assessed via flow cytometry.

B cell depletion

Early B cell depletion was performed 7 days prior to X-31 (H3N2) challenge. MD4 mice were injected intravenously via tail vein using a 29.5G syringe with 250 μg of Ultra-Leaf Purified anti-mouse CD20 Antibody (SA271G2, Biolegend, San Diego, CA) or with 250 μg of Rat IgG2b κ isotype control (RTK4530, Biolegend, San Diego, CA). Then

mice were oropharyngeally administered 125 µg of Ultra-Leaf Purified anti-mouse CD20 Antibody (SA271G2, Biolegend, San Diego, CA) or with 125 µg of Rat IgG2b κ isotype control (RTK4530, Biolegend, San Diego, CA). Late B cell depletion was performed intravenously and oropharyngeally with identical CD20 antibody and isotype volume and concentrations as early B cell depletion except treatment was performed 5 days (day 49) and 2 days (day 52) prior to PR8 infection (day 54) in WT (C57BL/6) mice.

Serum transfer experiments

WT or µMT mice were injected intravenously via tail vein with 150µL of PBS vehicle, pooled naïve serum collected from WT mice, or pooled influenza memory serum collected from WT mice with F/F/S infection at day of tissue harvest (day 61). Mice were passively immunized 6-7 hours following PR8 challenge (day 54).

Intravascular staining for flow cytometry

Mice were injected intravenously via tail vein using a 29.5G syringe with 100µg of PE anti-CD45 mAb (Biolegend, San Diego, CA) to label lymphocytes in the vasculature. Mice were euthanized three minutes after antibody injection. Mouse tissues were processed and stained for flow cytometry according to the methods outlined in *Flow Cytometry* except tissues were always kept in the dark immediately following dissection.

Bulk-RNA sequencing on Lung CD8$^+$ T cells

Mouse lung lobes were perfused with 2 mL of PBS and then were aseptically dissected and minced with sterile scissors and incubated in 10 mL of collagenase media

(DMEM; Life Technologies Fisher Scientific, Hampton, NH, Collagenase IV; Worthington Biochemical, Lakewood, NJ, DNase I; Sigma-Aldrich, St. Louis, MO) for 1 hour shaking at 37°C. After the incubation, the lungs were smashed through a 70-μm filter and rinsed with DMEM media with 10% fetal bovine serum (FBS) to obtain a single cell suspension. The single cell suspension was centrifuged (300g, 5 min). Cells were washed with PBS (10% FBS, 1mM $CaCl_2$), strained in a 40-μm filter, and centrifuged again. Cells were resuspended at a concentration of $1x10^8$ cells in PBS (10% FBS, 1mM $CaCl_2$). Dead cell removal was performed via negative selection using the EasySep Mouse Dead Cell Removal (Annexin V) kit (StemCell Inc., Vancouver, CA) as directed. The flow through was centrifuged and resuspended in PBS (10% FBS, 1mM EDTA) and $CD8^+$ T cells were isolated using the $CD8^+$ T cell Isolation Kit as directed (StemCell Incorporated, Vancouver, CA). RNA was extracted from isolated lung $CD8^+$ T cells using TRI reagent (Zymo Research, Irvine, CA). Samples were digested on ice for 5 minutes with mechanical agitation of samples occurring 2-3 times. RNA was further extracted from samples using the Direct-zol Mini Prep kit as directed. Samples were shipped to MedGenome (Foster City, CA) where library preparation (Takara SMART-Seq mRNA, San Jose, CA) and sequencing (Illumina NovaSeq, Oakdale, MN) were performed (100-bp single-end reads and 20 million reads per sample).

Bioinformatics

Bulk-RNA sequencing reads were aligned and analyzed by MedGenome (Foster City, CA). Gene Set Enrichment Analysis was performed using Webgestalt (WEB-based GEne SeT AnaLysis Toolkit) (80). Heatmaps were made using the provided TPM counts

and using R in R Studio version 4.1.0, data were $\log_2$-transformed and scaled according to row using the pheatmap package (81) to highlight expression levels for select genes across samples.

Statistics

Data were analyzed using GraphPad Prism software (San Diego, CA). Experiments were repeated two-six times as indicated. All data are presented as mean with SEM, unless otherwise noted. Mann-Whitney *U* test, one-way ANOVA with multiple comparisons, or two-way ANOVA were used for statistical significance with a p value ≤ 0.05.

Study Approval

All research with animal models was subject to prior review and approval and conducted in compliance by University of Pittsburgh's Institutional Animal Care and Use Committee (protocol #23073501).

Data availability

The datasets are available in the NCBI's Gene Expression Omnibus (GEO) repository (GEO GSE288913). Data are graphed as individual data points to depict experimental variation. All raw data will be provided in Supporting Data Values files on revision. Data are available upon request from the corresponding author, subject to institutional review and approval.

Target	Fluorophore	Clone	Manufacturer
CD4	Pacific Blue	RM4-5	BD Biosciences
CD8	AF532	53-6.7	ThermoFisher Scientific
CD8	BB700	53-6.7	BD Biosciences
CD45	BUV496	30-F11	BD Biosciences
CD45	PE	30-F11	Biolegend
CD90.2	BUV395	30-H12	BD Biosciences
TCR β	BB700	H57-597	BD Biosciences
B220	APC	RA3-6B2	BD Biosciences
B220	BUV496	RA3-6B2	BD Biosciences
CD19	BV605	1D3	BD Biosciences
CD11b	AF700	M1/70	ThermoFisher Scientific
CD11b	BV510	M1/70	Biolegend
CD11c	AF700	N418	Biolegend
CD11c	BUV395	N418	BD Biosciences
CD44	PerCP	IM7	Biolegend
CD62L	BUV737	MEL-14	BD Biosciences
CD103	BV480	2M90	BD Biosciences
CD103	BV711	2E7	Biolegend
GD TCR	BUV805	GL3	BD Biosciences
CD69	BV650	H1.2F3	Biolegend
PD1	PE-Cy7	RMP1-30	ThermoFisher Scientific
Tim3	BV421	RMT3-23	Biolegend
Tim3	BV711	RMT3-23	Biolegend
Lag3	BV785	C9B7W	Biolegend

Tbet	PE	4B10	Biolegend
Tbet	AF647	4B10	BD Biosciences
FOXP3	PE-Cy5	FJK-16s	ThermoFisher Scientific
GATA3	BV421	16E10A23	Biolegend
RORγT	PE-CF594	Q31-378	BD Biosciences
IFNγ	BV711	XMG1.2	Biolegend
IFNγ	PE	XMG1.2	BD Biosciences
IL-10	AF647	JES5-16E3	Biolegend
Perforin	PE-Dazzle594	S16009A	Biolegend
TNFα	PE-Dazzle594	MP6-XT22	Biolegend
Granzyme B	PE-cy7	QA16A02	Biolegend
CD64	BV650	X54-5/7.1	BD Biosciences
CD24	BV421	X54-5/7.1	Biolegend
F4/80	BV480	T45-2342	BD Biosciences
SiglecF	BB515	E50-2440	BD Biosciences
NK1.1	PE-cy5	PK136	Biolegend
Ly6c	BV785	HK1.4	Biolegend
Ly6g	APC-cy7	1A8	BD Biosciences
CD206	APC	C068C2	Biolegend
MHCII	BUV615	M5/114.15.2	BD Biosciences
CD80	BUV805	16-10A1	BD Biosciences
CD86	BUV737	GL1	BD Biosciences
CD40	PE-cy7	3/23	Biolegend
CD40	BV421	3/23	Biolegend
Arg1	AF700	A1exF5	ThermoFisher Scientific
iNOS	PE-efluor-610	CXNFT	ThermoFisher

			Scientific
KLRG1	PE-CF594	2F1	BD Biosciences
CX3CR1	BV711	SA011F11	Biolegend

Table 1 – Antibodies utilized for spectral flow cytometry.
